# Proteome of the Triatomine Digestive Tract: From Catalytic to Immune Pathways; Focusing on Annexin Expression

**DOI:** 10.3389/fmolb.2020.589435

**Published:** 2020-12-09

**Authors:** Marcia Gumiel, Debora Passos de Mattos, Cecília Stahl Vieira, Caroline Silva Moraes, Carlos José de Carvalho Moreira, Marcelo Salabert Gonzalez, André Teixeira-Ferreira, Mariana Waghabi, Patricia Azambuja, Nicolas Carels

**Affiliations:** ^1^Laboratório de Bioquímica e Fisiologia de Insetos, Instituto Oswaldo Cruz, Fundação Oswaldo Cruz (IOC/FIOCRUZ), Rio de Janeiro, Brazil; ^2^Research Department, Universidad Privada Franz Tamayo (UNIFRANZ), La Paz, Bolivia; ^3^Laboratório de Biologia de Insetos, Departamento de Biologia Geral, Universidade Federal Fluminense, Niterói, Brazil; ^4^Programa de Pós-Graduação em Ciências e Biotecnologia, Instituto de Biologia, Universidade Federal Fluminense, Niterói, Brazil; ^5^Departamento de Entomologia Molecular, Instituto Nacional de Entomologia Molecular (INCT-EM), Rio de Janeiro, Brazil; ^6^Laboratório de Doenças Parasitárias, Instituto Oswaldo Cruz, Rio de Janeiro, Brazil; ^7^Laboratório de Toxinologia, Instituto Oswaldo Cruz, Rio de Janeiro, Brazil; ^8^Laboratório de Genômica Funcional e Bioinformática, Instituto Oswaldo Cruz, FIOCRUZ, Rio de Janeiro, Brazil; ^9^Laboratório de Modelagem de Sistemas Biológicos, National Institute for Science and Technology on Innovation in Neglected Diseases (INCT-IDN), Centro de Desenvolvimento Tecnológico em Saúde (CDTS), Fundação Oswaldo Cruz (FIOCRUZ), Rio de Janeiro, Brazil

**Keywords:** chagas disease, triatomine, digestive tract, mass spectrometry, enzymes, immunity, annexin

## Abstract

*Rhodnius prolixus*, *Panstrongylus megistus*, *Triatoma infestans*, and *Dipetalogaster maxima* are all triatomines and potential vectors of the protozoan *Trypanosoma cruzi* responsible for human Chagas’ disease. Considering that the *T. cruzi*’s cycle occurs inside the triatomine digestive tract (TDT), the analysis of the TDT protein profile is an essential step to understand TDT physiology during *T. cruzi* infection. To characterize the protein profile of TDT of *D. maxima*, *P. megistus*, *R. prolixus*, and *T. infestans*, a shotgun liquid chromatography-tandem mass spectrometry (LC-MS/MS) approach was applied in this report. Most proteins were found to be closely related to metabolic pathways such as gluconeogenesis/glycolysis, citrate cycle, fatty acid metabolism, oxidative phosphorylation, but also to the immune system. We annotated this new proteome contribution gathering it with those previously published in accordance with Gene Ontology and KEGG. Enzymes were classified in terms of class, acceptor, and function, while the proteins from the immune system were annotated by reference to the pathways of humoral response, cell cycle regulation, Toll, IMD, JNK, Jak-STAT, and MAPK, as available from the Insect Innate Immunity Database (IIID). These pathways were further subclassified in recognition, signaling, response, coagulation, melanization and none. Finally, phylogenetic affinities and gene expression of annexins were investigated for understanding their role in the protection and homeostasis of intestinal epithelial cells against the inflammation.

## Introduction

Chagas disease (Chagas, 1909, 1911) is one of the major causes of acute myocarditis and progressive chronic cardiomyopathy in endemic regions of Latin America, affecting almost 6-7 million people (WHO (World Health Organization), 2020). The protist parasite *Trypanosoma cruzi* is the causative agent of Chagas disease and its development alternates between vertebrates (mainly mammals, but also lizards) and triatomine hosts (Garcia et al., 1999; Ferreira et al., 2016).

Triatomines are insects classified as Hemiptera: Reduviidae with some of the most important species for Chagas disease transmission being *Triatoma infestans, Rhodnius prolixus*, and *Panstrongylus megistus* (Coura and Dias, 2009; Schofield and Galvão, 2009; Coura, 2015). These triatomine species have wide geographical distribution in Latin America with marked adaptation to domestic and peridomestic ecotopes as well as physiological features that promote *T. cruzi* development (Guhl, 2007; Coura and Dias, 2009; Noireau, 2009; Gurgel-Gonçalves et al., 2012; Coura, 2015). By contrast, *Dipetalogaster maxima* is found in northern America, where it inhabits the sylvatic environments of Baja California Sur and Mexico. It resides in dry and rocky areas and usually takes its blood meal from lizards, but when fasting can bite human or domestic animals (Guzmán-Bracho, 2001).

Many factors influence the *T. cruzi* vectorial transmission, such as: (i) The varied feeding behavior of triatomines; (ii) The close association between triatomines and humans; (iii) The variety of ecotopes that triatomines occupy, which turns the control of their population difficult (Noireau et al., 2005; Cortez et al., 2007; Buitrago et al., 2010; Espinoza Echeverria et al., 2017); (iv) The wide phylogenetic divergence among the natural clones of *T. cruzi* (Laurent et al., 1997); and also (v) The geographic origin of *T. cruzi* and its vector that may constitute an essential factor in the parasitic cycle since local strains are usually better adapted to indigenous vector species than to exotic ones (Ryckman, 1965; Zeledón, 1987).

The life cycle of *T. cruzi* in the invertebrate host is restricted to the triatomine digestive tract (TDT) (Zeledón, 1987; Garcia et al., 1999; Gonzalez et al., 1999; Cortez et al., 2012; Dias et al., 2015; Ferreira et al., 2016). Therefore, *T. cruzi* suffers the influence of different factors present in the lumen of insect gut (Mello et al., 1996; Moreira et al., 2003; Azambuja et al., 2004, 2017; Garcia et al., 2010b; Castro et al., 2012; Nogueira et al., 2015; Vieira et al., 2016); these factors seem to differ constitutively according to triatomine species (Dworak et al., 2017; Guarneri and Lorenzo, 2017). The midgut of triatomines is divided into two portions: the anterior midgut (AM), where the blood is stored, and the posterior midgut (PM) where the protein digestion occurs (Billingsley and Downe, 1983, 1986a).

Since the midgut is a natural barrier for resistance to foreign pathogens, the identification of proteins in the TDT is an essential step toward the determination of their role in *T. cruzi* interaction with insect epithelial cells and the immune system. Therefore, TDT proteomic analysis could allow a better understanding of the processes involved in (i) blood digestion, (ii) nutrient absorption, (iii) *T. cruzi* adhesion to digestive tract surfaces following by multiplication or differentiation, as well as (iv) the local humoral defense mechanisms associated to the intestinal bacterial microbiota after triatomine feeding (Alves et al., 2007; Nogueira et al., 2007; Gonzalez et al., 2011, 2013; da Mota et al., 2012; Oliveira et al., 2012; Vieira et al., 2016; Nogueira et al., 2017; Moreira et al., 2018).

To briefly summarize the molecular knowledge that has been recently acquired on TDT, Ribeiro et al. (2014) identified thousands of genes regularly expressed, which were further recognized among the 15,546 putative genes reported by Mesquita et al. (2015) in the genome of *R. prolixus.* Later on, immune-related genes were detected in *Triatoma pallidipennis*, *T. dimidiata*, and *T. infestans*, using comparative transcriptomics based on gene references from the immune response of hexapods (Zumaya-Estrada et al., 2018). These studies show that the repertoire of genes in the immunological signaling pathway is substantially different in Hemiptera compared to Diptera. Since the methodology based on genomic sequences needs to be complemented by the direct analysis of gene products through functional genomics, a proteome analysis was, first, conducted by 2D gel electrophoresis by Vieira et al. (2015) using the AM of *R. prolixus* and, later on, by Ouali et al. (2020) using both AM and PM. These authors mainly found proteins involved in detoxification, amino acids, lipids and sugar degradation; they also confirmed the existence of these proteins by reference to the transcriptome and genome annotations by Ribeiro et al. (2014) and Mesquita et al. (2015).

In complement to sequence analyses, Antunes et al. (2013) investigated the fecal metabolome of *P. megistus*, *R. prolixus* and *T. infestans* expanding the knowledge of the TDT chemical composition. These authors reported that 80% of the metabolites found were common to these three species, while the remaining 20% varied among them (Antunes et al., 2013).

Here, we aimed at investigating the complete proteomic profile of the TDT from four species: *T. infestans*, *R. prolixus*, *P. megistus*, and *D. maxima* to take a step forward in the knowledge of *T. cruzi* vectors biology. The shotgun proteome results described here seek to contribute to the understanding of the factors that may influence the triatomine competence for transmitting Chagas disease.

Through shotgun proteomic approach (nano-LC/MS/MS), we identified proteins expressed in midgut epithelial cells and rectum enriched fraction of the TDT from the four triatomine species analyzed in this study. We found that the main functions activated in the TDT are biased toward energy production, with most enzymes associated with the citrate cycle, glycolysis, and fatty acid metabolism. Another noticeable contribution of this report regards the annotation of proteins associated with the insect immune system. We found a significant contribution of putative superoxide dismutases, catalases, serine proteinases, heat shock proteins related to JAK/STAT as well as other proteins associated with Toll and IMD pathways.

In the context of the search for proteins possibly implicated in the triatomine immune system, we also detected three annexins (described initially in *R. prolixus*) in our proteome samples. After comparing these three annexins with sequences from Ribeiro et al. (2014), Vieira et al. (2015) and Ouali et al. (2020) through *Basic Local Alignment Search Tool* (BLAST) analyses, we noted that these authors already found them confirming previous inferences from the complete genome sequence. Annexins are a family of proteins that are associated with many biological events, including calcium-binding, interaction with membranes, intracellular vesicle trafficking, arachidonic acid release, leukocyte migration, and that also affects several mediators involved in the inflammatory response including cyclo-oxygenase-2 (Cox-2) and inducible nitric oxide synthase (Minghetti et al., 1999; Ferlazzo et al., 2003; Rescher and Gerke, 2004; Gavins and Hickey, 2012). Annexins presents a highly variable amino-terminal domain, possibly resulting in distinct functions specific to the members of a given family (Gerke and Moss, 2002; Moss and Morgan, 2004; Rescher and Gerke, 2004). Annexins are conserved among eukaryotes (Gerke and Moss, 2002) and have been described in many organisms since the unicellular eukaryote *Giardia* (Raynal and Pollard, 1994) to humans (Gerke and Moss, 2002; Lizarbe et al., 2013). The structure and function of annexins were poorly described in insects. A way to shed light on the annexins in insects and, more particularly, in triatomines, which is the interest here, is to look at them from the perspective of their evolution. Phylogenetic analyses and gene expression experiments were performed here in an attempt to improve the discussion concerning the role of annexins in triatomines.

The main contribution of this study was to show that the groups of catalytic and immune proteins reported by Ouali et al. (2020) were respectively formed by (i) enzymes involved in the energy metabolism and (ii) proteins from the humoral response, cell cycle regulation, Toll, IMD, JNK, Jak-STAT, and MAPK. Within the pathways of immune response, we described the expression profile of annexins that are believed to play an important role in inflammation processes elicited by antigens derived from the commensal microbiota that may interact with the *T. cruzi* biology.

## Materials and Methods

### Triatomine Breeding

Fifth instar *R. prolixus* nymphs were obtained from a colony maintained at the *Laboratório de Bioquimica e Fisiologia de Insetos* at 28°C ± 2°C and 60–70% relative humidity as described by Azambuja and Garcia (1997). Insects were fed on defibrinated rabbit blood through a membrane feeding apparatus. *D. maxima*, *P. megistus*, and *T. infestans* fifth instar nymphs were collected from a colony of the *Laboratório de Doenças Parasitárias* from the Instituto Oswaldo Cruz. These triatomine species were fed on live chicken. Two specimens of *D. maxima*, *P. megistus*, and *R. prolixus* and three *T. infestans* were chosen randomly from 15 to 21 days post-feeding for experiments.

### Digestive Tract Preparation

Triatomines were dissected by cutting the connective membrane laterally and taking the dorsal cuticle out with sterilized forceps using a stereoscopic microscope model Motic Q766 (Quimis, Diadema, SP, Brazil) at 12x magnification. Immediately, each digestive tract, including AM, PM, and rectum, was opened, and washed three times with sodium phosphate buffer (PBS) to remove blood content. Afterward, digestive tracts were collected in sterile microcentrifuge tubes and immediately preserved on ice until the addition of lysis buffer (50 mM Tris–HCl, pH 8, 150 mM NaCl, 0.1% SDS, 1% NP-40, 0.5% Deoxycholate, 1 mM CaCl_2_) and a cocktail of protease and phosphatase inhibitors (P8340-Sigma) in a 1:100 proportion. Lysis was performed through two to three short ultrasound pulses of 10 s each with a Misonic sonicator XL-2000 (QSonica LLC) and each tube was maintained in ice. All steps were performed under aseptic conditions.

### Protein Extraction

Protein precipitation was performed according to Cascardo (Cascardo et al., 2001) with some modifications. Each sample was added to ice-cold acetone containing 17% TCA (w/v), and homogenized. After precipitation for 20 min at −20°C, the mixture was centrifuged at 15,000 *g* for 5 min at 4°C. After the elimination of the supernatant the pellet was then rinsed three times with ice-cold acetone/TEA (triethanolamine) 2% before an additional step of centrifugation at 15,000 *g* for 30 min at 4°C. Each pellet was resuspended in an isoelectric focusing buffer (2% CHAPS, 8 M Urea) and stored at -80°C. Protein concentration was determined by the RCDC method (BioRad), using bovine serum albumin as standard.

100 μg of proteins of each sample were mixed with 20 μL of 400 mM ammonium bicarbonate and 8 M urea and were reduced by incubation in 10 μL of 100 mM dithiothreitol for 3 h at 37°C. After cooling to room temperature, each sample was alkylated with 10 μL of 400 mM iodoacetamide for 15 min in the dark. The urea concentration was diluted to 1 M, following the addition of 280 μL deionized water. Trypsin (Promega, San Luis Obispo, CA, United States) was added at an enzyme/substrate ratio of 1:50 (w/w) and the digestion process was performed for 17 h at 37°C. The reaction was interrupted with the addition of 40 μL of 10% (v/v) formic acid to a final concentration of 1%. The number of independent biological replicates were two in the case of *D. maxima*, *P. megistus*, and *R. prolixus* and three in the case of *T. infestans*. The digested peptide mixture was desalted and concentrated using MacroSpin C18 columns (The NestGroup, Southborough, MA). Peptides were finally eluted with 0.1% formic acid in 50% v/v acetonitrile, completely dried in a vacuum centrifuge and then suspended in 20 μL of 1% formic acid. For each sample, peptides from 10 μL desalted tryptic peptide digests were separated into a 15 cm (75 μm internal diameter) column packed with 3 μm 200A ReproSil-Pur C18-Qrix (Dr. Maisch, Germany). Chromatography was carried out on an EASY-nLC II (Thermo Scientific, United States). The mobile phase A consisted of 0.1% (v/v) formic acid in water, while the mobile phase B consisted of 0.1% (v/v) formic acid in acetonitrile, with gradient conditions of 5 to 40% B for 164 min em up to 80% B in 2 min, and remains at 80% for 2 min.

### Protein Identification by Mass Spectrometry

Eluted peptides were directly introduced to a linear trap quadrupole (LTQ) Orbitrap XL ETD (mass spectrometry facility RPT02A/Oswaldo Cruz Institute, FIOCRUZ, Rio de Janeiro) for analysis. Mass spectra were acquired in a positive mode using the data-dependent automatic (DDA) survey MS scan and tandem mass spectra (MS/MS) acquisition. Each DDA consisted of a survey scan in the m/z range 300–1,700 and a resolution of 60,000 with a AGC target value of 1 × 10^–6^ ions. The 7 most intense peaks obtained in MS1 were subjected to collision-induced fragmentation (CID) in the ion trap analyzer LTQ with minimal signal required 1 × 10^–4^, using the collision-induced dissociation (CID) with normalyzed collition energy in 35, and previously fragmented ions were dynamically excluded for 45 s. The spray voltage was 1.9 kV, 100 μA current, 48 V capillary voltage, 200°C capillary temperature and 99.9 V lens tube voltage.

### Data Analyses

The raw files (technical triplicates for each of the samples) produced by LTQ-Orbitrap with the list of MS2 fragmentation peptides were analyzed using MaxQuant (vs 1.6.3.3, Cox and Mann, 2008). These raw files were compared to a list of 68,875 protein sequences extracted from the UniprotKB server^1^ including *R. prolixus* (14,941 protein genes), *Acyrthosiphon pisum* (pea aphid, 35,819 protein genes), and *Diaphorina citri* (Asian citrus psyllid, 21,517 protein genes), which were the only completely annotated genomes of Hemiptera available at the time of the study. We though that the comparison to several protein genes of Hemiptera’s species could compensate for enventual error or missing data of gene annotations in *R. prolixus*. Raw MS files, the list of parameters settled on Maxquant, and also text files generated by MaxQuant analysis were up-loaded via MASSIVE HPC facility^2^ (Goscinski et al., 2015) and deposited in the ProteomeXchange Consortium^3^ under the accession numbers: PXD021625 for *D. maxima*, PXD021626 for *P. megistus*, PXD021627 *R. prolixus*, and PXD021628 for *T. infestans*.

The list of proteins with accession numbers (TrEMBL, version 09, 2018) from mass spectrum analyses (proteinGroups.txt from MaxQuant) were pooled together and freed from redundancies. Accession samples from each species were analyzed for inter-species redundancies before depiction with InteractiVenn (www.interactivenn.net, Heberle et al., 2015).

To integrate the proteome of this study to previous knowledge, we gathered the sequences from our proteome samples with the datasets of *R. prolixus* from Ribeiro et al. (2014), Vieira et al. (2015) retrieved from the *Bioinformatics Resource for Invertebrate Vectors of Human Pathogens* (VectorBase,^4^), and Ouali et al. (2020) from ProteomeXchange Consortium. We retrieved the equivalent of UniprotKB accession considering an identity region (BLASTp) of 70% of the query sequence with a similarity ≥ 80% in the case of sequences from Ribeiro et al. (2014) and Vieira et al. (2015). Since the sequences from Ouali et al. (2020) were already given with UniprotKB accessions, this exercise was not necessary. To sum up the gene onthologies (GO terms; Ashburner et al., 2000) of non-redundant protein sequences of TDT (Supplementary File 1) collected until now (*n* = 3,736; Supplementary File 1a) through the proteome analyses quoted above, we used ClueGO (v2.5.7; Bindea et al., 2009) whitin Cytoscape (v3.8.0; Shannon et al., 2003) with the default parameters (except for pV that was set to 0.5 given the limitation of ClueGO to analyze less than 1,473 vertices) and importing the *R. prolixus* dataset. To focus on GO terms associated to enzymatic function, we first retrieved the sequences associated to *Enzyme Commission numbers* (EC) from the prokaryotes and eukaryote accessions of the *Kyoto Encyclopedia of Genes and Genomes* (last free version of KEGG from 2015, https://www.genome.jp/kegg/). Second, we transfered (BLASTp) EC terms (subject) to proteome sequences (query) when the best alignment corresponded (expected value ≤ 0.0001) to a similarity region of at least 70% of the query sequence and an identity level ≥ 60% (the final accession list was *n* = 1,060; Supplementary File 1b). Third, we annotated these enzyme sequences with GO terms using ClueGO. To investigate enzymatic function according to KEGG pathways, we had to decrease the threshold level for function transfer to its limit of significancy, i.e., identity level ≥ 60% over an identity region of at least 40 amino acids, given the low level of identity between triatomine and KEGG sequences. To improve pathway depiction, we deleted the non-relevant alternative routes of the most completed pathways.

### Innate Immune System

We also annotated the sequences of our samples and those of Ribeiro et al. (2014), Vieira et al. (2015) and Ouali et al. (2020) for their involvement in the immune system by comparison (BLASTp) with the *Insect Innate Immunity Database* (IIID, http://bordensteinlab.vanderbilt.edu/IIID/test_immunity.php) (Brucker et al., 2012), transferring function from the *subject* to the *query* sequence following the same criteria as described above in the comparison of proteins with KEGG. In a second time, we updated the information relative to “Toll and Imd signaling pathways” (code: 04624) as well as “MAPK signaling pathway” (code: 04013) with the data of immune pathways from KEGG^5^. For MAPK, we only focused on the insect proteins that participate in the wound healing, immune response, and ROS production responses since the other routes of the map number 04013 are not directly related to the immune system.

PGRPs are proteins involved in the digestive and immune system (Kaneko et al., 2006; Royet and Dziarski, 2007; Broderick, 2015; Van Niekerk and Engelbrecht, 2015); thus, we also downloaded the protein sequences corresponding to the search keyword “PGRP” from the *National Center for Biotechnology Information* (NCBI,^6^) server. However, we only included insects in this search by selecting this category from the “Results by taxon” menu. We also included three *R. prolixus* annexin sequences (RPRC013832; RPRC011897; RPRC003519) in our analysis since they have a crucial role in triatomine immune system.

Similarly to the previous analysis of the metabolic pathway, the best alignment for function transfer was chosen to be with an expected value of less than or equal to 0.0001, an identity greater than or equal to 60% in a region of at least 40 amino acids.

### Sequences Identification of *R. prolixus* Annexins

Annexins were retrieved from VectorBase and their protein sequences were analyzed by comparison with those retrieved from the Non-redundant Protein Sequence (*nr*) and Reference Protein (*refseq*) sections from NCBI as well as from the UniProtKb/Swiss–Prot^7^ and Protein Data Bank (RCSB PDB, https://www.rcsb.org/) servers using BLASTp.

### Phylogenetic Analyses of *R. prolixus* Annexins

Neighbor-Joining (NJ) phylogenetic trees for annexins were constructed using protein sequences from insects, vertebrates, and fungus. Annexin amino acid sequences used in phylogenetic analyses were from (i) *Homo sapiens* (P04083, P07355, P07355-2, P12429, P09525, P09525-2, P08758, P08133, P08133-2, P20073, P20073-2, P13928, P13928-2, P13928-3, O76027, Q9UJ72, P50995, P50995-2, P27216, and P27216-2), *Pediculus humanus* (E0VHI3, E0VUL0, E0V9K1, and E0VM42), *Glossina morsitans morsitans* (A0A1B0G8D6, A0A1B0G9I8, A0A1B0FD10, and D3TLB6), *Drosophila melanogaster* (P22464, P22464-2, P22464-3, A0A0B4KH34, P22465, Q9VXG4, Q9VXG4-2, and Q9VXG4-3), and *Anopheles gambiae* (F5HJB3, A0A1S4GJ86, Q5TVB3, F5HJB1, F5HJB2, Q7PS96, Q7QG24, and Q5TVB0), which were retrieved from UniprotKB; (ii) *Rhodnius prolixus* (RPRC011897-RA, RPRC003519-RA, and RPRC013832-RA) retrieved from VectorBase; (iii) *Aspergillus spp.* fungus (XP_747470.1, EAW15291.1, AAK61604.1, and GAO81894.1); *Blastomyces gilchristii* (XP_002629554.1); *Saprolegnia monoica* (ABC59142.1); *Phytophthora infestans* (AID48672.1) retrieved from NCBI; and (iv) *Diplocarpon rosae* (BUE80_DR013296, BUE80_DR004611) retrieved from *EnsemblFungi^8^*.

The tree was constructed using MEGA-X version 10.0.5 (Kumar et al., 2018), according to the NJ statistical method with (i) Poisson model with uniform rates among sites and pairwise deletions, and (ii) bootstrap values set to 10,000 replications, as parameters.

### Gene Expression of *R. prolixus* Annexins

The expression of *R. prolixus* annexin genes (RPRC011897, RPRC003519, and RPRC013832-RA) was investigated through *real-time quantitative polymerase chain reactions* (RT-qPCR). The specific primers for RT-qPCR were designed using both Primer3 and Beacon Designer. The specificity of primers was verified *in silico* by comparison with the *R. prolixus* genome sequence available at VectorBase using BLAST. The primers design is detailed in Table 1. Primers for *R. prolixus* housekeeping genes (α*-tubulin* and *GAPDH*) were designed as previously described (Paim et al., 2012).

**TABLE 1:** Primer sequences for annexin detection.

Annexin types	VectorBase accessions	Primer types	Up	Sequence	Down
1	RPRC011897	Forward	5′	CGTAGTTACCAACACCTGAGACAG	3′
1	RPRC011897	Reverse	5′	GAAGACCATCCTTAATGCTACCCG	3′
2	RPRC003519	Forward	5′	CGGTGCTGGTACGAAAGATAGAG	3′
2	RPRC003519	Reverse	5′	CTTCCTCCAAAGTCCTGCCATAC	3′
3	RPRC013832	Forward	5′	CGAACGTCTAGAAGACAGTATGGCAC	3′
3	RPRC013832	Reverse	5′	CTCCGGCACAATCATTAGCGATACG	3′

AM and PM samples from *R. prolixus* fifth instar nymphs were dissected at day one and day seven after feeding (DAF) to collect and separate midgut samples in three pools containing five AM or PM each (Vieira et al., 2016). Total RNA extraction and quantification were performed as described in Vieira et al. (2016) using the NucleoSpin^®^ RNA II Kit (Macherey-Nagel, Düren, Germany) and the NanoDrop 2000 Spectrophotometer (Thermo Scientific, Waltham, MA, United States), respectively. cDNA was synthesized with a First-Strand cDNA Synthesis Kit (GE Healthcare, Buckinghamshire, United Kingdom) using 2.5 μg of total RNA and the pd(N)6 primer. A Quantus Fluorimeter (PROMEGA) was used to quantify the cDNA with the QuantiFluor ssDNA System (PROMEGA). GoTaq^®^ qPCR Master Mix (PROMEGA) was used to perform RT-qPCR, measured in an ABI PRISM 7500 Sequence Detection System (Applied Biosystems) at the Fiocruz facilities (Real-Time PCR Platform RPT-09A). All RT-qPCR were performed using the following parameters: initial denaturation at 95°C for 20 s, 40 cycles at 95°C for 3 s and one cycle at 60°C for 30 s. Melting curves were performed to confirm that only one amplicon was amplified for each pair of primers. In all experiments of RT-qPCR, RPRC011897 was named as RpAnnexin1, RPRC003519 was named as RpAnnexin2, and RPRC013832 was named as RpAnnexin3. The results were analyzed as described by Vieira et al. (2016). Concerning the relative quantification of annexins, the calibration varied according to the comparison type. When comparing expression levels between midgut compartments (AM vs. PM), we set the expression of AM samples to 1 and gave the expression values of annexins in PM as fold changes of AM. When we compared the annexin expression between different days after feeding, in each midgut compartment, we set to 1 the expression values recorded in samples from 1 DAF, both from the AM or PM and gave the expression at 7 DAF as fold changes of 1 DAF.

After RT-qPCR, aliquots of reactions were cleaned up using Illustra^TM^ GPX^TM^ PCR DNA and Gel Band Purification Kit (GE Healthcare, Buckinghamshire, United Kingdom). For improvement of purifications of these samples, some protocol adaptations were included, such as two washing steps with Wash Buffer and a centrifugation step at 16,000 × *g* for 2 min after the last wash as well as the amplicon elution by adding nuclease-free water (50 μL) followed by 15 min incubation and a final step of centrifugation at 16,000 × *g* for 2 min. Purified amplicons were sequenced in a 96-capillaries ABI3730xl (Applied Biosystems) at the Fiocruz facilities (Sequencing Platform RPT-01A).

### Ethics Statement and Biodiversity Rights

*Rhodnius prolixus* (Hemiptera: Reduviidae) were obtained from a long-standing colony reared in the laboratory at 28°C ± 2°C and 60–70% relative humidity (Azambuja and Garcia, 1997). The other triatomine species (*T. infestans*, *P. megistus*, and *D. maxima*) used in this study are from the insectary of the *Laboratório de Doenças Parasitárias* from the *Instituto Oswaldo Cruz*. These insects were fed weekly on chickens and raised as previously described (Perlowagora-Szumlewicz and Moreira, 1994) according to the *Ethical Principles in Animal Experimentation* approved by the *Ethics Committee in Animal Experimentation* (CEUA/FIOCRUZ) under protocol number P-54/10-4/LW12/11. This protocol is from CONCEA/MCT^9^, which is associated with the *American Association for Animal Science* (AAAS), the *Federation of European Laboratory Animal Science Associations* (FELASA), the *International Council for Animal Science* (ICLAS) and the *Association for Assessment and Accreditation of Laboratory Animal Care International* (AAALAC). Genetic biodiversity property was authorized under numbers 13659-9 by the *System of Authorization and Information in Biodiversity* (SISBIO) and AE65C23 by *National Management System Genetic Heritage and Associated Traditional Knowledge* (SISGEN) of the *Brazilian Ministry of Environment*.

## Results

### Shotgun Proteome

**FIGURE 1:**
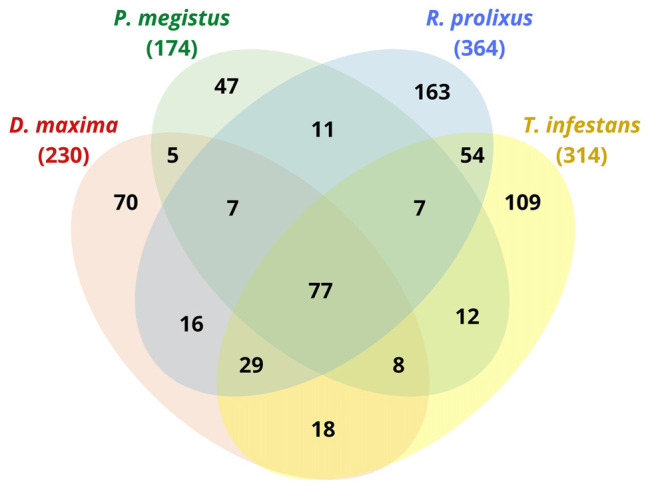
Venn diagram of proteins found in the digestive tract of *D. maxima*, *P. megistus*, *R. prolixus*, and *T. infestans.*

In our shotgun analysis, we identified 364, 230, 174, 314 (total = 1,082) non-redundant protein groups for *R. prolixus*, *D. maxima*, *P. megistus*, and *T. infestans*, respectively, whose molecular weights ranged from less than 15 kDa up to more than 90 kDa and isoelectric points (pI), varying from 4 to 11 (data not shown). When comparing species between them through Venn diagram, we found redundancies as displayed in Figure 1. Thus, rather than a total of 1,082, we found 633 non-redundant proteins considering the four species that are distributed as shown in Figure 1.

### Protein Groups

Integrating our shotgun analysis (*n* = 633) to the proteomes of Ribeiro et al. (2014), Vieira et al. (2015) and Ouali et al. (2020), we obtained a non-redundant list of 3,736 UniprotKB accessions. Among the ontogeny of *Biological Processes* (Supplementary File 2), ClueGO generated a topological network (Supplementary File 2a) of 22,291 edges based on 2,569 vertices with a high degree of connectivity. In particular, this network was characterized by a completely connected component associated to *proteins-containg complex assembly* (11.58%, connected to the heterogeneous groups of *cellular component biogenesis* and *cellular protein-containg complex assembly* at the interface with *translation process*). The two other major Biological Processes of this network were cellular amide metabolic process (14.15%, connected to the heterogeneous groups of *DNA-binding transcription factor activity* and *translation process*) and *transmembrane transporter activity* (14.47%, connected to the heterogeneous groups of *oxoacid metabolic process* and *organic acid metabolic process*), both with high connectivity levels forming well separated modules. Two other significant modules were formed by *mitochondrion* (5.14%, *organelle membrane* and *generation of precursor metabolites and energy*) and *amide transport* (4.82%, connected to the heterogeneous groups of *membrane coat*), both groups were interconnected via the process of *membrane protein complex*. *Guanyl nucleotide binding* and *endopeptidase complex* formed isoladed modules. The fine distribution of genes per GO term is given in the form of a histogram in the Supplementary File 2b and the statistics in Supplementary File 2c. Every ClueGO data files supporting Supplementary File 2a–c are available in Supplementary File 2d.

In terms of *Cellular Components* (Supplementary File 3), ClueGO generated a topological network (Supplementary File 3a) of 6,405 edges based on 1,306 vertices with a high degree of connectivity as well. This network was characterized by a completely connected component associated to *membrane coat* (15.91%, connected to *whole membrane* at the interface with *organelle membrane* and *mitochondrion –* 13.64%). Statistics showed that this network accounted for two other major groups, which were *membrane protein complex* (12.5%, connected to *mitochondrion*) and *non-membrane-bounded organelle* (9.09%, connected to *ribosome*). *Organelle lumen*, *ribosomal subunit*, *intracellular organelle*, *endopeptidase complex* and *eukaryotic translation initiatiation factor 3 complex* formed isoladed modules. The fine distribution of genes per GO term is given in the form of a histogram in the Supplementary File 3b and the statistics in Supplementary File 3c. Every ClueGO data files supporting Supplementary File 3a–c are available in Supplementary File 3d.

The topological network generated by ClueGO in case of *Molecular Functions* (Supplementary File 4) accounted for 795 nodes and 2,087 edges (Supplementary File 4a). Three major components were found in this network: channel activity (21.95%), guanyl ribonucleotide binding (17.07%), and intra molecular oxidoreductase activity, interconverting aldoses and ketoses (4.88%). The fine distribution of genes per GO term is given in the form of a histogram in the Supplementary File 4b and the statistics in Supplementary File 4c. Every ClueGO data files supporting Supplementary File 4a–c are available in Supplementary File 4d.

To better understand the enzymes’ contribution to the combined proteome, we did the same ClueGO exercise, but only considering proteins associated to EC numbers (*n* = 1,060, i.e., 28.4% of the total proteome). Considering *Biological Processes* (Supplementary File 5) again, ClueGO generated a topological network (Supplementary File 5a) of 8,756 edges based on 703 vertices with a large completely connected components and 12 smaller ones. The major ones was dedicated to *phosphorus metabolic process* (19.8%, connected to *purine* and *nucleotide/ribonucleotide metabolic processes* at the interface with *carbohydrate derivative metabolic process*, *carbohydrate metabolic process*, and *organic acid metabolic process* and connected to *adenyl ribonucleotide binding* and *phosphorus metabolic process*). The next five larger processes were about *carboxylic acid metabolic process* (8.05%), *organic acid metabolic process* (7.38%), *cellular amino acid metabolic process* (7.38%, connected to *alpha-amino acid metabolic process*), *adenyl ribonucleotide binding* (7.38%), and *cellular catabolic process* (6.71%, connected to *small molecule catabolic process* and *organic substance catabolic process*). *Guanyl nucleotide binding* and *endopeptidase complex*, *response to toxic substance*, *transaminase activity*, and *mitochondrion* formed isoladed modules. The fine distribution of genes per GO term is given in the form of a histogram in the Supplementary File 5b and the statistics in Supplementary File 5c. Every ClueGO data files supporting Supplementary File 5a are available in Supplementary File 5d.

In the case of *Cellular Components* (Supplementary File 6), we obtained a network of 175 vertices and 798 edges (Supplementary File 6a). The vertex groups of this network were all independent and were *mitochondrion* (29.63%), *proton-transporting two-sector ATPase complex* (29.63%), *proteasome complex* (18.52%), and *intracellular organelle* (7.41%). The fine distribution of genes per GO term is given in the form of a histogram in the Supplementary File 6b and the statistics in Supplementay File 6c. Every ClueGO data files supporting Supplementary File 6a–c are available in Supplementary File 6d.

Finally, when considering the *Molecular Function* (Supplementary File 7), the network obtained was made of 383 vertices and 3,044 edges (Supplementary File 7a). It was made of three main components: *guanyl ribonucleotide binding* (18.75%), *nucleotide binding* (15.62%), *transaminase activity* (7.81%), and *oxidoreductase activity, acting on the CH-OH groups of donors, NAD or NADP as acceptor* (7.81%). The fine distribution of genes per GO term is given in the form of a histogram in the Supplementary File 7b and the statistics in Supplementay File 7c. Every ClueGO data files supporting Supplementary File 7a–c are available in Supplementary File 7d.

Aiming at a better comprehension of how the functions outlined above may participate to the TDT metabolism, we mapped them on the KEGG pathways.

### Metabolic Pathway and Enzyme Annotations

**FIGURE 2:**
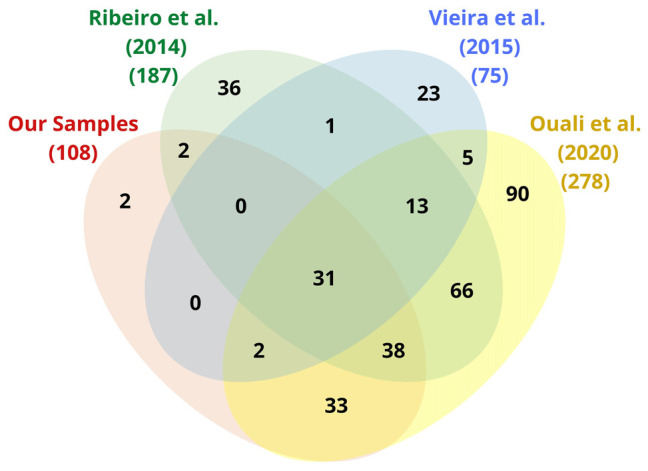
Venn diagram (Interactivenn) of complete EC annotations from our samples and those of Ribeiro et al. (2014), Vieira et al. (2015), and Ouali et al. (2020). The total number of enzyme functions (ECs) is indicated beside each ellipse.

We mapped 108, 187, 75, and 278 complete ECs as well as 10, 28, 5, and 34 incomplete ECs in our samples, and those of Ribeiro et al. (2014), Vieira et al. (2015), and Ouali et al. (2020), respectively. When removing the redundancies between datasets, we computed 342 complete and 38 partial ECs (Supplementary File 8). Among the complete ECs, only 2 ECs from our samples were neither present in Ribeiro et al. (2014), Vieira et al. (2015), nor Ouali et al. (2020; Figure 2).

Enzymes can be differentiated in seven classes (first EC number) according to the type of function they deserve. In each class, every enzyme is characterized by a four-digit code the three last of which represent a progressively finer classification with the last describing the full enzymatic reaction. A same EC number may correspond to more than one protein sequence, but these proteins perform the same enzymatic function. Thus, the numbers of annotated proteins that had enzymatic function in the present report, in Ribeiro et al. (2014), in Vieira et al. (2015), and Ouali et al. (2020) were 188, 366, 96 and 575, respectively (Table 2). The most representative classes were hydrolases, oxidoreductases and transferases, being associated with more than 73% of all enzymes identified in the four studies.

**TABLE 2:** Number of proteins and complete ECs distributed by enzyme classes.

Enzymatic Class	This work^1^		Ribeiro et al. (2014)		Vieira et al. (2015)		Ouali et al. (2020)	
				
	Proteins (%)	ECs (%)	Proteins (%)	ECs (%)	Proteins (%)	ECs (%)	Proteins (%)	ECs (%)
**Hydrolases**	57 (30.3)	22 (20.4)	99 (27.0)	43 (23.0)	33 (34.4)	21 (28.0)	175 (30.4)	58 (20.9)
**Oxidoreductases**	54 (28.7)	37 (34.3)	74 (20.2)	50 (26.7)	19 (19.8)	16 (21.3)	116 (20.2)	66 (23.7)
**Transferases**	31 (16.5)	22 (20.4)	95 (26.0)	56 (30.0)	20 (20.8)	21 (28.0)	149 (25.9)	82 (29.5)
**Translocases**	15 (8.0)	5 (4.6)	31 (8.5)	7 (3.7)	7 (7.3)	3 (4.0)	39 (6.8)	8 (2.9)
**Lyases**	13 (6.9)	8 (7.4)	15 (4.1)	13 (7.0)	8 (8.3)	7 (9.3)	26 (4.5)	19 (6.8)
**Isomerases**	8 (4.3)	6 (5.5)	25 (6.8)	11 (5.9)	7 (7.3)	5 (6.7)	28 (4.9)	14 (5.0)
**Ligases**	10 (5.3)	8 (7.4)	27 (7.4)	7 (3.7)	2 (2.1)	2 (2.7)	42 (7.3)	31 (11.2)
**TOTAL**	188 (100)	108 (100)	366 (100)	187 (100)	96 (100)	75 (100)	575 (100)	278 (100)

*The sequences of our samples matching UniprotKB and the sequences from the datasets of *R. prolixus* from Ribeiro et al. (2014), Vieira et al. (2015), and Ouali et al. (2020) retrieved from VectorBase and ProteomeXchange were annotated for their enzymatic function (EC number) by reference to KEGG using BLASTp. ^1^The sum *D. maxima, P. megistus, R prolixus* e *T. infestans* samples.*

**FIGURE 3:**
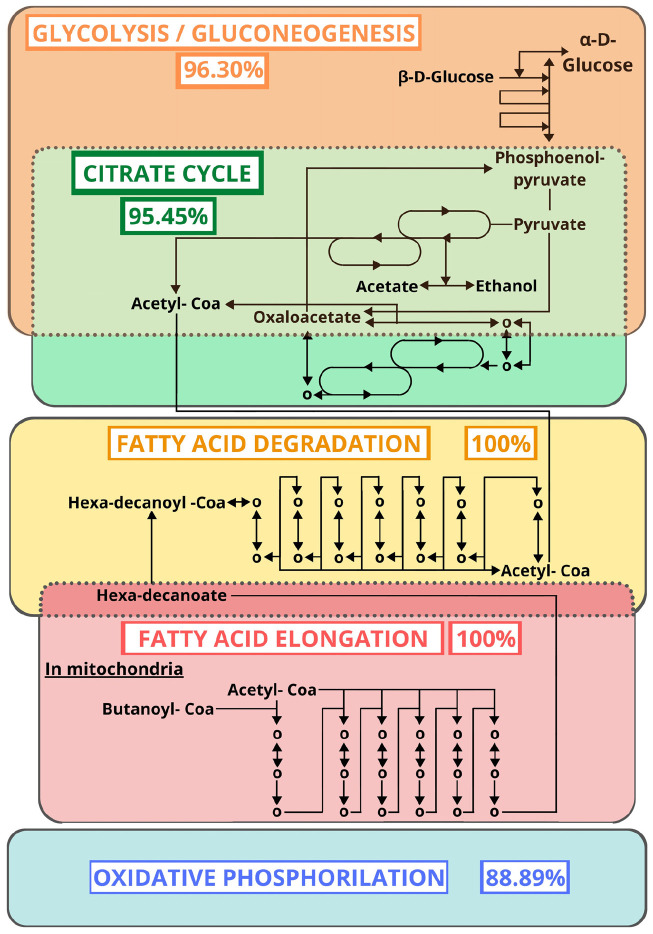
Metabolic pathways (KEGG) with the highest proportion of mapped ECs after excluding alternative routes. The percentages represent the proportion of ECs found by similarity search.

Despite variations, the protein and EC distributions of Table 2 were rather consistent, which suggests the coherence of our results with previous reports. The enzymatic functions reported in Figure 2 and Table 2 were mapping to 106 KEGG metabolic pathways. By similarity comparison (BLASTp) of the proteins of our sample sequences with the insect section of KEGG (1.1.1.192, 1.6.5.11, 2.3.1.61, 2.7.4.1, 3.6.1.1, 4.1.1.1, 1.2.1.48, 2.3.1.12, 2.7.1.99, 3.1.2.22, 3.6.3.10, 6.2.1.6, CPT1, and CPT2), we succeeded in filling in most gaps in these pathways. We found that those pathways with the highest proportion of mapped ECs, after excluding alternative routes (Figure 3), were citrate cycle (95.45%; Supplementary File 9), fatty acid elongation (100%; Supplementary File 10), fatty acid degradation (100%; Supplementary File 11), glycolysis/gluconeogenesis (96.30%; Supplementary File 12), and oxidative phosphorylation (88.89%; Supplementary File 13). These results suggest that the primary function of the TDT is to produce energy from digestion (Figure 3).

In addition to these pathways, we found several enzymes linked to the immune system, such as catalase (EC:1.11.1.6, two proteins), superoxide dismutase (EC:1.15.1.1, three proteins), peroxidase (EC:1.11.1.7, three proteins), glutathione transferase (EC:2.5.1.18, one protein), and phospholipase A2 (EC:3.1.1.4, ten proteins) (Supplementary File 8). The peroxiredoxins and thioredoxins found in our samples are peroxidases that can reduce hydroperoxides (Hofmann et al., 2002). These enzymes are able to protect *Anopheles stephensi* and *Drosophila melanogaster* cells against oxidative stresses (Peterson and Luckhart, 2006; Radyuk et al., 2010). Catalases, are other antioxidant enzymes that were observed in the digestive tract of the four triatomine species analyzed here, which suggests their involvement in the intestinal epithelium protection against hydrogen peroxide and the oxidative stress generated by blood digestion (Oliveira et al., 2011; Ouali et al., 2020). The accumulation of ROS, which can be produced abruptly as part of blood digestion, promotes the action of several enzymes, such as superoxide dismutase, glutathione transferase, as a mechanism of detoxification already identified in hematophagous insects such as mosquitoes and *R. prolixus* (Paes et al., 2001; DeJong et al., 2007; Nogueira et al., 2015). Some other gut detoxification enzymes, such as sulfotransferase is present in our analysis as well. They were also described in Ribeiro et al. (2014).

### Innate Immune System

As outlined in the previous section, we found enzymes involved in the immune system, which promoted us to investigate proteins from the innate immune system of triatomines present in our samples as well as in those of Ribeiro et al. (2014), Vieira et al. (2015), and Ouali et al. (2020). We believe that these proteins can be important to understand the compatible relationship between triatomines and *T. cruzi*.

In our samples, we found 25 proteins associated with 22 genes from immune pathways. By contrast, in Ribeiro et al. (2014) we found 22 proteins associated with 25 genes, and in Vieira et al. (2015) we found 9 proteins associated with 12 genes from immune pathways. Finally in Ouali et al. (2020) we found 57 proteins associated with 57 genes from immune pathways. These genes participate in seven different pathways, which were humoral response, cell cycle regulation, Toll, IMD, JNK, Jak-STAT, and MAPK. These pathways were further classified as recognition, signaling, response, coagulation, melanization or none of these options (Table 3). The functions associated with these pathways have been described in 57 insect species (Supplementary File 14).

**TABLE 3:** Pathways of the innate immune system from triatomines.

Associated Pathway	Class	Related genes			
		This work^1^	Ribeiro et al. (2014)	Vieira et al. (2014)	Ouali et al. (2020)
**None**	**−**	1	0	0	1
**Cell Cycle**	**Cellular Response**	0	0	0	1
**Humoral Response**	**Coagulation**	1	1	1	2
**Humoral Response**	**Response**	2	4	4	4
**Humoral Response**	**Melanization**	0	0	0	1
**IMD**	**Recognition**	0	2	0	2
**IMD**	**Signaling**	0	2	0	5
**IMD**	**−**	0	0	0	1
**IMD and MAPK**	**Response**	0	1	0	0
**IMD and MAPK**	**Signaling**	0	2	0	2
**JAK/STAT**	**Response**	8	1	5	11
**JAK/STAT**	**Signaling**	1	0	0	1
**JAK/STAT, JNK and Cell Cycle**	**Signaling**	0	0	0	2
**JAK/STAT, JNK and Cell Cycle**	**−**	0	0	0	1
**JNK**	**Signaling**	0	0	0	1
**MAPK**	**Signaling**	0	2	0	6
**Toll**	**Recognition**	1	0	0	0
**Toll**	**Response**	0	1	0	0
**Toll**	**Signaling**	1	2	0	1
**Toll and IMD**	**Signaling**	0	0	0	3
**Toll, IMD and MAPK**	**Signaling**	0	0	0	1
**Unknown**	**−**	5	7	2	9
**Unknown**	**Response**	2	0	0	2
**TOTAL**		22	25	12	57

*The protein sequences of our samples and those from the datasets of *R. prolixus* from Ribeiro et al. (2014), Vieira et al. (2015), and Ouali et al. (2020) were then annotated by reference to IIID, the immune pathways from KEGG (code: 04624 and 04013), insects’ PGRP proteins, RPRC003519, RPRC011897 and RPRC013832 sequences from NCBI, using BLASTp. ^1^The sum *D. maxima, P. megistus, R prolixus* e *T. infestans* samples.*

**FIGURE 4:**
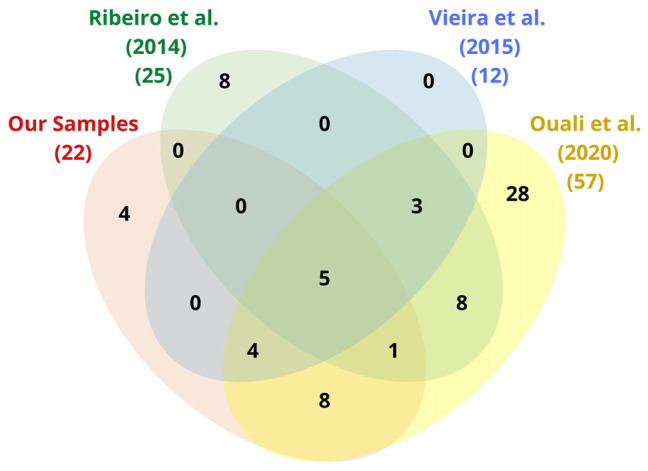
Venn diagram of the immune-related genes found by mapping the sequences of our triatomine samples as well as those of Ribeiro et al. (2014), Vieira et al. (2015), and Ouali et al. (2020) by reference to IIID, KEGG, and NCBI (see Table 3).

After the removal of redundancies between samples, a total of 69 related genes were computed for the innate immune system with 4 of them neither present in Ribeiro et al. (2014) nor in Vieira et al. (2015), nor in Ouali et al. (2020; Figure 4).

### Sequences Identification of *R. prolixus* Annexins

The immune control of many intracellular pathogens, including *T. cruzi*, was reported to be dependent on the production of nitric oxide (Ascenzi and Gradoni, 2002). In mice, a higher susceptibility to *T. cruzi* infection is associated to the reduction of inducible nitric oxide synthase (iNOS) gene expression, nitric oxide (NO) and γ-interferon (IFN-γ) levels (Felizardo et al., 2018). Nitric oxide (NO) is released during the acute phase of *T. cruzi* infection in mice and treatment with inhibitors of NO synthase exacerbates the infection (Vespa et al., 1994; Hölscher et al., 1998). Annexin regulation is associated with nitric oxide synthase (NOS) induction by bacterial lipopolysaccharide in macrophages (Wu et al., 1995; Minghetti et al., 1999; Ferlazzo et al., 2003; Rescher and Gerke, 2004; Gavins and Hickey, 2012). Nitric oxide (NO) is known to have a protective effect on the gastrointestinal tract. In preclinical studies NO was shown to help maintain gastric mucosal integrity, to inhibit leukocyte adherence to the endothelium, and to repair damages induced by anti-inflammatory drugs (Lanas, 2008). Inflammatory conditions of the gastrointestinal tract are a significant cause of morbidity in vertebrates and ANXA1-deficiency decreases the repair of intestinal mucosal injury (Babbin et al., 2008; Sena et al., 2013). Thus, it seems reasonable to think that the modulation of annexin synthesis could be part of the pathogenesis process of *T. cruzi* in triatomines.

Considering that *R. prolixus* is the triatomine species whose genome sequencing is the most advanced, we choose it to analyze the gene expression of annexins. The genome of *R. prolixus* accounts for only three annexins: RPRC011897 (RpAnnex1), RPRC003519 (RpAnnex2), and RPRC013832 (RpAnnex3). We retrieved three sequences with similarity to annexins, as shown in Table 4.

**TABLE 4:** Best hits of annexins with different databases using BLASTp.

Data Base	Description of Best hit	Query Cover,%	Identities (%)	E-value	Accession number
**RpAnnex1 RPRC011897**					
Non-redundant Protein Sequence (*nr*)	Annexin B9 isoform X2 [*Halyomorpha halys*]	99.0	270/321 (84)	0.0	XP_014289507.1
Reference Protein (refseq)	Annexin B9 isoform X2 [*Halyomorpha halys*]	99.0	270/321 (84)	0.0	XP_014289507.1
InsectBase	[*Zootermopsis nevadensis*]	98.8	245/319	0.0	KDR23347.1
			(77)		
Trembl (Insects)	[*D. melanogaster*]	99.7	237/322	e-176	tr| A0A0B4KH34
			(73)		
UniProtKb/Swiss -Prot	Annexin B9	99.0	227/322	4e-165	P22464.2
	[*D. melanogaster*]		(70)		
RBC PDB	Chain A, Annexin A4	96.0	161/311	2e-107	2ZOC_A
	[*Homo sapiens*]		(52)		
UniProtKb/Swiss -Prot	[*Homo sapiens*]	96.0	161/311	e-106	sp| 09525
	Annexin A4		(52)		
**RpAnnex2 RPRC003519**					
InsectBase	[*Zootermopsis nevadensis*]	102.0	282/513 (55)	3.55e-170	KDR15476.1
Trembl (Insects)	[*P. humanus*]	91.5	241/461 (52)	e-158	tr| E0V9K1
UniProtKb/Swiss -Prot	[*Homo sapiens*] Annexin A11	63.5%	167/320 (52)	e-107	sp| P50995
**RpAnnex3 RPRC013832**					
InsectBase	[*Zootermopsis nevadensis*]	98.7	213/317 (67)	6.11e-152	KDR08631.1
Trembl (Insects)	[*Anopheles gambiae*]	99.7	242/320 (59)	e-138	Tr| F5HJB1
UniProtKb/Swiss -Prot	[*Homo sapiens*] Annexin A11	99.7	156/311 (50)	7e-98	sp| P50995

### Phylogenetic Analyses of *R. prolixus* Annexins

**FIGURE 5:**
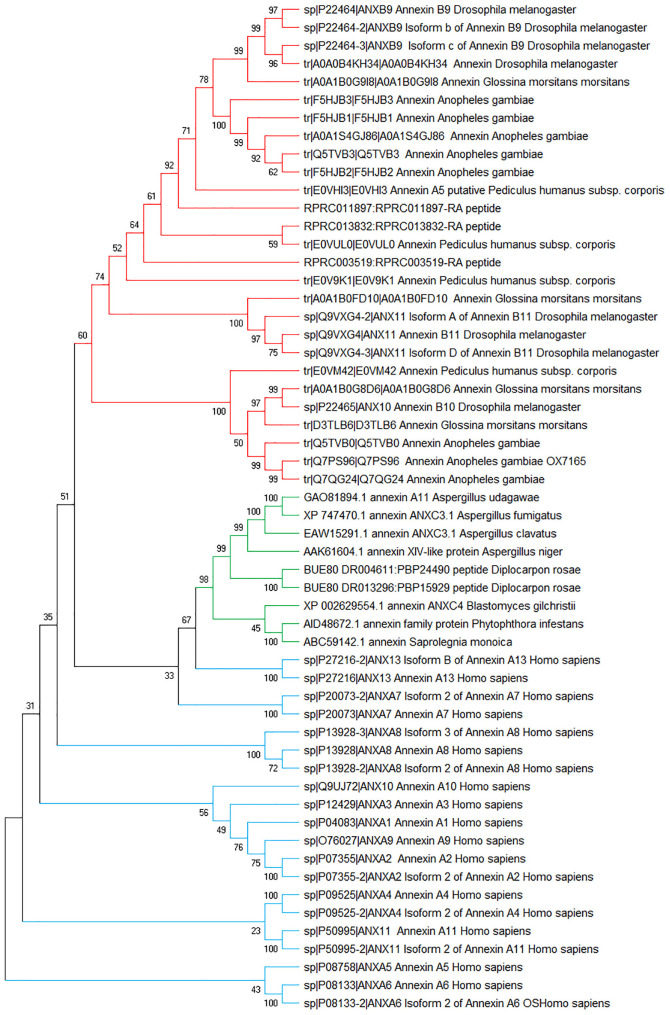
Phylogenetic tree of annexin sequences from fungi, insects, and humans. Non-rooted NJ phylogenetic tree was constructed with MEGA-X4. The numbers adjacent to the branches represent bootstrap values based on 10,000 replicates.

The phylogenetic analysis showed that insect and fungal annexins clustered in different clades (Figure 5, red branch for insect annexin, green branch for fungi), forming monophyletic groups. Also, we found that the human annexins are more diversified than the other annexins from fungi and insects, and unlike these both groups, human sequences of annexins have not clustered in a unique group (Figure 5, blue branches). Furthermore, human annexins A13 (ANXA13) and A7 (ANXA7) grouped with fungal annexins at the root of the insect clade, suggesting a common annexin ancestor in the fungal clade.

Within *R. prolixus*, the three annexins clustered rather coherently, in the insect branch and curiously, RPRC013832 (RpAnnex3) clustered with *P. humanus*. However, the structure of the tree is not well supported by bootstrap values, which is not surprising due to the evolutive distance between *R. prolixus* and *P. humanus*.

Despite the sequence conservation (BLASTp similarity between *R. prolixus* and *H. sapiens* comparisons extended over 77% to 100% of sequence extention), the functionality could not be transferred from human to *R. prolixus* given the low (below 60%) similarity levels (RPRC013832 vs ANXA11: 50%; RPRC011897 vs ANXA4: 51%; RPRC003519 vs ANXA7: 46%).

### Gene Expression of *R. prolixus* Annexins

In order to confirm that the three annexin proteins detected here could be functional in triatomines, we studied the expression of these annexin genes based on their genome sequences in *R. prolixus*. Here, we confirm the differential expression of the three annexin genes between the first and the seventh day after blood ingestion in the anterior and posterior midgut from *R. prolixus*. These two midgut compartments have differences according to their roles in blood storage and digestion (Terra and Ferreira, 1994), distinct degrees of immune responses as well as density of microbiota populations (Castro et al., 2012; Ribeiro et al., 2014; Vieira et al., 2016).

**FIGURE 6:**
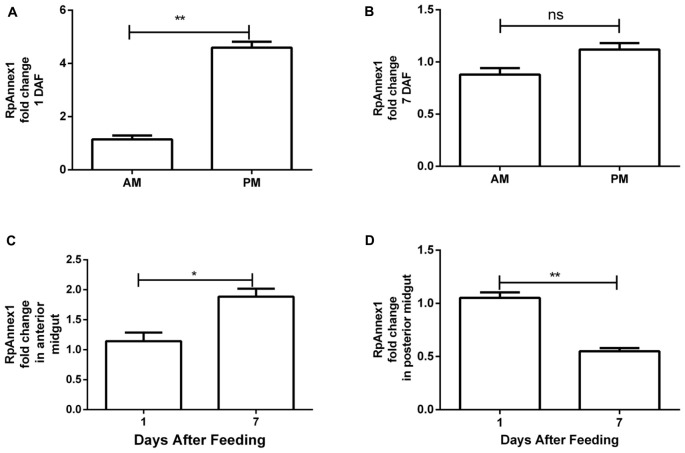
Spatial and temporal relative gene expression of RPRC011897 (RpAnnex1) in fifth instar nymphs of *R. prolixus*. Blood-fed insects were dissected, and the digestive tract was separated in anterior midgut (AM) and posterior midgut (PM) at 1 and 7 days after feeding (DAF). **(A)** Expression of RpAnnex1 in AM and PM at 1 DAF. **(B)** Expression of RpAnnex1 in AM and PM at 7 DAF. **(C)** Expression of RpAnnex1 in AM at 1 and 7 DAF. **(D)** Expression of RpAnnex1 in PM at 1 and 7 DAF. Calibrators genes used in the relative quantification of gene expression were α*-tubulin* and *GAPDH*. Bars represent the mean ± the standard error of the mean (SEM) of two independent experiments with three pools of *R. prolixus* (*n* = 3). Means were compared applying Student’s *t*-test; **p < 0.01, *p < 0.05, and ns stands for non-significant.

For assessing annexin expression, we performed RT-qPCR assays on AM and PM samples of *R. prolixus* individuals at 1 and 7 DAF. We found that the expression of the RPRC011897 gene (RpAnnexin1) was much larger in PM than in AM at 1 DAF (*p* < 0.01, Figure 6A). However, despite the same trend, the difference of RpAnnexin1 expression between AM and PM was not significant at 7 DAF (*p* > 0.05, Figure 6B). When analyzing the expression of RpAnnexin1 gene in AM and PM separately at 1 DAF and 7 DAF, we found a significant increase of this gene expression in AM from the 1st DAF to the 7th DAF (*p* < 0.05, Figure 6C) while the expression of this same gene decreased in PM in the same interval of time (*p* < 0.01, Figure 6D). In other terms, annexin 1 responded faster to blood intake in PM than in AM; it increased first in PM and 7 days later, it increased in AM.

**FIGURE 7:**
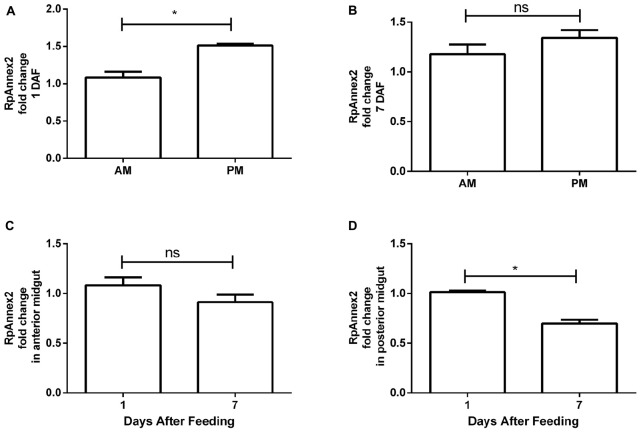
Spatial and temporal relative gene expression of RPRC003519 (RpAnnex2) in fifth instar nymphs of *Rhodnius prolixus*. Blood-fed insects were dissected, and tissues were separated in anterior midgut (AM) and posterior midgut (PM) at 1 and 7 days after feeding. **(A)** Expression of RpAnnex2 in AM and PM at 1 day after blood feeding, **(B)** Expression of RpAnnex2 in AM and PM at 7 day after blood feeding, **(C)** Expression of RpAnnex2 in AM at 1 and 7 days after blood feeding, **(D)** Expression of RpAnnex2 in PM at 1 and 7 days after blood feeding. Calibrators genes used in the relative quantification of gene expression were α*-tubulin* and *GAPDH*. Bars represent the mean ± the standard error of the mean (SEM) of two independent experiments with three pools of *R. prolixus* (*n* = 3). Means were compared applying Student’s *t*-test; *p < 0.05, and ns stands for non-significant.

The expression profile of RPRC003519 (RpAnnexin2) gene was similar to that of the RpAnnexin1 gene, showing higher expression in the PM at 1 DAF than in the AM (*p* < 0.05, Figure 7A), but at a lower extend. Again, despite the same trend, the difference of RPRC003519 expression in the AM and PM at 7 DAF (*p* > 0.05, Figure 7B) was not significant. In contrast to the RpAnnexin1 gene, there was no difference in the expression of RpAnnexin2 gene in AM at 1 and 7 DAF (*p* > 0.05, Figure 7C), however, the expression of this gene was significantly higher in the PM at 1 DAF (*p* < 0.05, Figure 7D). In other terms, both annexins 1 and 2 were expressed at a higher level in PM compared to AM at 1 DAF, however this trend was much more significant for annexins 1 than for annexin 2.

**FIGURE 8:**
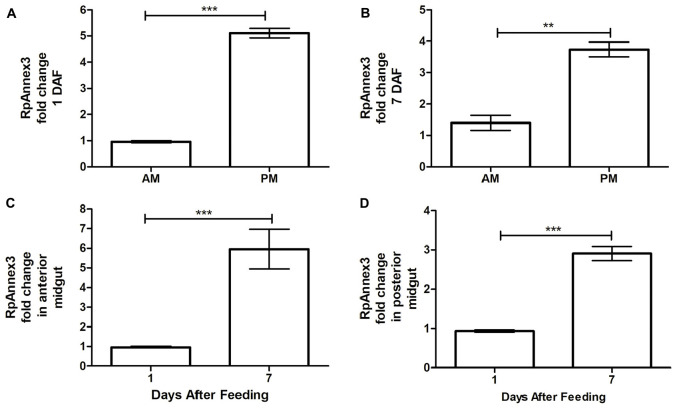
Spatial and temporal relative gene expression of RPRC013832 (RpAnnex3) in fifth instar nymphs of *Rhodnius prolixus*. Blood-fed insects were dissected and tissues were separated in anterior midgut (AM) and posterior midgut (PM) at 1 and 7 days after feeding. **(A)** Expression of RpAnnex3 in AM and PM at 1 day after blood feeding, **(B)** Expression of RpAnnex3 in AM and PM at 7 day after blood feeding, **(C)** Expression of RpAnnex3 in AM at 1 and 7 days after blood feeding, **(D)** Expression of RpAnnex3 in PM at 1 and 7 days after blood feeding. Calibrators genes used in the relative quantification of gene expression were α*-tubulin* and *GAPDH*. Bars represent the mean ± the standard error of the mean (SEM) of two independent experiments with three pools of *R. prolixus* (*n* = 3). Means were compared applying Student’s *t*-test; **p < 0.01 and ***p < 0.001.

Finally, we found that the expression of RPRC013832 (RpAnnexin 3) was higher by as much as more than three times in PM than in AM at both 1 and 7 DAF (*p* < 0.001, Figure 8A; *p* < 0.01, Figure 8B). Comparing the RpAnnex3 expression in each midgut compartment, we could demonstrate that this transcript was more abundant at 7 DAF than at 1 DAF in both AM and PM samples (*p* < 0.001, Figure 8C; *p* < 0.01, Figure 8D).

In other terms, RpAnnex3 was upregulated on the seventieth day after blood ingestion but is more abundant in the PM than in the AM.

## Discussion

Triatomines ingest blood at all nymphal stages and can reach up to 12 times their body weight after a single repast (Schofield, 1980). During the digestion process, the availability of nutrients promotes the growth of cultivable bacteria that reach a peak of growth on the 7th day after blood ingestion by *R. prolixus* (Azambuja et al., 2004; Castro et al., 2012; Vieira et al., 2016). In this study, we used triatomines from the fifth instar nymph three weeks after feeding at which time the populations of bacteria are low (Castro et al., 2012).

The bottom-up methodology of proteome characterization that we used here, also called shotgun, includes the tryptic digestion of complex protein solutions and their peptide fractionation by liquid chromatography, followed by mass spectrometry (MS). The obtained results are fragments of a whole and, although it is possible to identify a protein-based on its peptides, a peptide can be lost during the chromatography or not generate suitable mass spectra (Armirotti and Damonte, 2010). Some enzymes were not always present in the four-triatomine species analyzed. Differences in observed protein samples could be due to the variations among the species related to the amount of food ingested, digestive process, or even sampling heterogeneity.

### Protein Ontologies

The homeostasis of all living cells depends on the balance between metabolism and gene regulation. Like all other animals, insect development, growth, and other physiological processes are coordinated in response to tissue metabolic status or environmental cues which activate gene expression mechanisms (Tortoriello et al., 2013; Van der Knaap and Verrijzer, 2016). The primary way of gene regulation requires signal transduction pathways that control key transcription factors orchestrating gene expression and protein synthesis (Ferrandon et al., 2007). Additionally, different metabolic cues from the extracellular environment are sensed by different transmembrane cell receptors (Marinho et al., 2014; Van der Knaap and Verrijzer, 2016). Besides, post-translational modifications of chromatin play a substantial role in the activation or repression of gene transcription. These processes include acetylation, methylation, and phosphorylation of the histones as well as DNA methylation. Acetylation of histones is mediated by histone acetyltransferases, enzymes that use acetyl-CoA as substrate (Shogren-Knaak et al., 2006; Van der Knaap and Verrijzer, 2016). Among the ontogeny of Biological Process, acetyl-CoA metabolic process is classified in cellular amide metabolic process.

In this sense, our results pointed out a significant amount of proteins related to cellular amide metabolic processes in triatomine digestive tract, which includes several enzymes involved in the transcription factors activity and post-translational modifications. Also, transmembrane transport activity enzymes were abundant in our analysis, essential to transmit signals from extracellular midgut content to the epithelial cells (Ferrandon et al., 2007). Triatomine gut is an environment that suffers oxidative stress and microbiota proliferation after a blood meal ingestion (Paim et al., 2016; Vieira et al., 2020). The metabolites derived from digestion or from microbiota could act as a signal or substrate to some insect enzymes that could interfere in molecules or proteins from signaling pathways, increasing or repressing gene transcription, maintaining gut homeostasis (Schroeder et al., 2008; Marinho et al., 2014; Van der Knaap and Verrijzer, 2016; Cohen et al., 2017).

Morphologically, perimicrovillar membranes (PMM) cover the plasma (microvillar) membrane (MM) of the midgut epithelium apical surface of hematophagous triatomines (Lane and Harrison, 1979; Andries and Torpier, 1982; Billingsley and Downe, 1986b). Once PMM form a net on the midgut, one expect a major association between membrane coat and cellular components as was observed in midgut proteome. The organelle membrane also stands out on data analysis due to PMM being formed by lipoproteins that leave the endoplasmic reticulum of the epithelial cells and form double membrane vesicles seen budding from some Golgi areas (Lane and Harrison, 1979; Andries and Torpier, 1982; Billingsley and Downe, 1988; Silva et al., 1995). These vesicles can also contain some protein as hydrolytic digestive enzymes (i.e., peptidases) (Billingsley and Downe, 1983, 1985; Ferreira et al., 1988; Terra et al., 1988, 2006; Silva et al., 1995), which justify the membrane protein complex and ribosome association to major cellular components and the proteins-containing complex assembly to biological processes network.

The mitochondria role in this tissue is in agreement with the subsequent analyses in which we observed that the most evident metabolic pathways are related to the production of energy, also highlighted in the ClueGO analyses.

The active flux of ions in the midgut is assumed to be related to the absorption of organic substances, like amino acids by appropriate protein carriers present in PMM. In the perimicrovillar space (between PMM and MM), amino acids may diffuse into midgut cells by specific transporters – as potassium ion-amino acids co-transporters – on the microvillar surface (Ferreira et al., 1988; Albuquerque-Cunha et al., 2009). This channel activity was associated to main molecular functions in proteome data.

### Metabolic Pathway and Enzyme Annotations

Here, proteins involved in protein digestion such as, cathepsin L and D were identified in the different triatomines species. Cathepsin D is induced in the different portions of the midgut of *T. infestans* (Balczun et al., 2012) and its activity has been demonstrated to increase approximately by 3-fold in *R. prolixus* infected with *T. cruzi* (Buarque et al., 2013; Borges et al., 2006). We corroborated the observation of cathepsin D made by Balczun et al. (2012) in *T. infestans* gut in the fifth day after a blood meal. These results indicate that the knowledge about the enzyme activities involved in the digestion process is essential to elucidate their kinetics after blood meal. Other factors, such as the amount of blood ingested as well as the blood source should also be considered.

Most proteins related to the metabolism of carbohydrates such as glyceraldehyde 3-phosphate dehydrogenase (Kunieda et al., 2006), fructose-bisphosphate aldolase, phosphopyruvate hydratase, triose-phosphate isomerase, alcohol dehydrogenase, phosphoglycerate kinase, aldehyde dehydrogenase, and phosphoglycerate mutase, were identified here, which is not surprising since digestive tract is the place for extracting energy and converting small molecules from food.

Acyl-Coa dehydrogenase is a key enzyme in lipid metabolism (He et al., 2011) and was identified in all four triatomine species of this report. Other enzymes associated with lipid metabolism were identified as well, which is not surprising since fatty acid metabolism enzymes are actively involved in energy fueling as it is the case for the carbohydrate metabolism enzymes. There are indications that triatomines predominantly use their metabolism to extract energy from fat sources to fuel their molting process when the energy demand is intense.

### Antioxidant and Stress Proteins

Regarding blood digestion, hemoglobin (Hb) as well as peptides, amino acids, and heme proteins, are also released into the TDT (Francis et al., 1997). Besides the digestion process, the volume of blood consumed by triatomines determines the limit of their nymphal development (Kollien et al., 2001; Salvati et al., 2001, 2002) as well as the metacyclogenesis of *T. cruzi* (Garcia et al., 1995; Lander et al., 2020). In its free state, the heme molecule acts as a potent pro-oxidant and cytotoxic agent, leading to the lysis of many cells and ROS generation through the catalytic decomposition of organic hydroperoxides (Van der Zee et al., 1996; Graça-Souza et al., 2006). The generation of ROS promotes oxidative stress that can oxidize disulfide bridges and lead to protein unfolding resulting in their loss of activity (Schieber and Chandel, 2014). The participation of chaperones such as disulfide isomerase, identified in *D. maxima* and *T. infestans* (T1HJ96), can mitigate the oxidative damage caused by the oxidation in disulfide bridges. Disulfide isomerase performs an essential role in the rearrangement of disulfide bridges to promote correct protein folding and preventing their aggregation (Freedman et al., 2017).

### Innate Immune System

The TDT is also a primary site of interaction with natural microbiota and functions as a barrier against pathogens ingested by insects (Wigglesworth, 1936; Garcia et al., 2010a). Conserved signaling pathways orchestrate the synthesis of effector molecules after an immune challenge (Ferrandon et al., 2007; Buchon et al., 2009a, b; Imler, 2014). In this sense, the mapping of immune proteins in the TDT is a crucial step for a better understanding of the biological processes related to insect development and their interaction with their natural parasites and bacterial microbiota (Ursic-Bedoya and Lowenberger, 2007; Zumaya-Estrada et al., 2018).

Proteins related to canonical immune signaling pathways (Toll, IMD and JAK/STAT) were identified in all triatomine species studied here. The AMP defensin were found in our analysis from the data of Ribeiro et al. (2014). Defensins are antimicrobial peptides known for their action against Gram-positive bacteria (Ganz, 2003; Lopez et al., 2003; Vieira et al., 2014) and to be up-regulated in *T. brasiliensis* and *R. prolixus* in response to infection by *T. cruzi*, which demonstrates a role of defensin in parasite control (Araújo et al., 2006; Waniek et al., 2011; Vieira et al., 2016).

Heat shock proteins were identified in the different species of this study. The HSP70 family were previously described in the *R. prolixus* digestive tract (Ribeiro et al., 2014; Mesquita et al., 2015) and were also associated with the response to temperature stress in both *D. melanogaster* and *R. prolixus* (Krebs and Feder, 1998; Paim et al., 2016). The expression of *hsp70* is considered a useful marker for inducible stress response in an organism and it was demonstrated that some elicitors induced by stress activate the JAK/STAT pathway by increasing the translocation of STAT and upregulating the *hsp70* gene expression and proteins synthesis (Madamanchi et al., 2001; Bettencourt et al., 2008; Colinet et al., 2010). Interestingly, the heat shock protein 90 kDa has chaperone activity, which may aid in the folding of polypeptides produced under stress conditions (Timperio et al., 2008). Triatomines might be confronted with various long-term challenging factors, which could promote *hsp70* up-regulation, including predation (Hawlena and Schmitz, 2010), climate changes (Chown and Nicolson, 2004; Paim et al., 2016), parasitism (Chang, 2005), and blood ingestion (Paim et al., 2016). Heat shock proteins seem to play a significant role in various physiological processes in triatomines. It was suggested that *hsp70* is vital for resistance to starvation in *T. infestans* (Kollien and Billingsley, 2002). Additionally, it was verified that the knockdown of *hsp70* genes in *R. prolixus* induces downregulation of lysozymes, relish and IMD gene expression, suggesting a role of HSP70 in the modulation of triatomine immune system (Paim et al., 2016). In addition, it also indicates a possible connection between JAK/STAT and the IMD pathway in *R. prolixus*.

Besides the HSP functions discussed above, *hsp* genes are expressed under many other stressful circumstances such as osmotic dysregulation, hypoxia, recognizing of the component of a microbial cell wall, physical injury and oxidative stress (Wojda, 2017). HSPs bind aberrant nuclear and cytosolic proteins to protect them against their denaturation and irreversible forwarding to the ubiquitin-proteasome system for degradation. The physiological role of ubiquitins in triatomine is still an open question so far. However, their presence in the midgut proteomic of *R. prolixus* analyzed here from the data of Ouali et al. (2020), demonstrates their significance. It is known that in Diptera, ubiquitination reaction is associated with virus infection and apoptosis (Schreader et al., 2003; Troupin et al., 2016).

The concept of inflammation in insects is controversial, but several publications demonstrated that fundamental elements of the inflammatory responses are conserved in insects and mammals (Pastor-Pareja et al., 2008; Stanley et al., 2009; Jiravanichpaisal et al., 2010; Razzel et al., 2011; Bangi, 2013; Weavers et al., 2018). Inflammation is a primordial response that protects the host against microbial infection and injury caused by exposure to xenobiotics or even by blood digestion in hematophagous insects (Graça-Souza et al., 2006; Perretti and D’Acquisto, 2009; Lee and Lee, 2014). However, an augmented response could lead to harmful consequences to the organism (Dennis and Norris, 2015). During an inflammatory response, some endogenous anti-inflammatory pathways are activated to down-regulate and maintain this response under control (Kamal et al., 2005; Khan et al., 2017). Therefore, the idea of anti-inflammation has recently been established to illustrate the balance that exists between pro- and anti-inflammatory mediators that work in concert to initiate, maintain, and finally resolve the inflammatory reaction, vital for restoring tissue structure and homeostasis (Perretti and D’Acquisto, 2009; Dennis and Norris, 2015). Inflammation in insect gut could be caused by tissue injury due to microbial infection, the release of oxidant reagents, or even by microbiota proliferation (Ferrandon, 2013; Lee and Lee, 2014; Lestradet et al., 2014; Lee et al., 2016; Charroux et al., 2018). During mucosal inflammation, the secretion of anti-inflammatory mediators is a mechanism that is critical in controlling inflammatory responses and promoting epithelial restitution and barrier recovery (Babbin et al., 2008; Lee and Lee, 2014).

### *R. prolixus* Annexins

The JAK/STAT pathway has been described to have a role in insect inflammation as well as in gut regenerative process (Buchon et al., 2009a, b; Jiang et al., 2009; Herrera and Bach, 2019), and some proteins related to this signaling pathway were detected in the present work, as discussed above. In *Drosophila*, components of JAK/STAT pathway are homologous to inflammatory mediators from mammals (Rose-John, 2018; Herrera and Bach, 2019). However, the role of JAK-STAT in inflammation mechanisms in triatomines remains obscure and needs further investigation. On the other hand, the eicosanoid pathway, that regulates pro-inflammatory mediators, have been studied in several insects (Stanley et al., 2009; Stanley and Kim, 2019) including in the triatomine *R. prolixus* (Garcia et al., 2004; Figueiredo et al., 2008). In this context, we emphasized the annexins, which are proteins that were described to play a critical immune regulatory role in inflammation in several organisms (Hannon et al., 2003; Babbin et al., 2008; Perretti and D’Acquisto, 2009) and were found to be expressed in the proteome described here. So far, annexins were poorly studied in triatomines. They have poor affinities with those of common models of insects, but insects clearly clustered altogether in a clade separated from those of fungi and human. Taking fungi as the common ancestor, insects and vertebrates seems to stem from ANXA13, which is consistant with Braun et al. (1998). The low bootstrap values between insect and human annexins prohibit any functional extention of human annexins to the insects’ ones. This observation is supported by low similarity levels between both groups and the emergence of a large number of isoforms in human (*n* = 12) compared to only three in *R. prolixus*. For this reason, we believe that annexins should be studied for their own in insects.

In vertebrates, annexins A1 are up-regulated by glucocorticoids and ANXA1 suppresses phospholipase A_2_ (PLA2), thereby blocking eicosanoid production (Parente and Solito, 2004; Bandorowicz-Pikula et al., 2012). Glucocorticoids inhibit prostaglandins and leukotrienes, the two main products of inflammation, at the level of PLA2 as well as at the level of cyclooxygenase/PGE isomerase (COX-1 and COX-2; Goppelt-Struebe et al., 1989), which potentiate the anti-inflammatory effect (Raynal and Pollard, 1994; Mira et al., 1997; Rao, 2007). Moreover as outlined above, annexin regulation is associated with NOS induction by bacterial lipopolysaccharide in macrophages (Minghetti et al., 1999; Ferlazzo et al., 2003; Rescher and Gerke, 2004; Gavins and Hickey, 2012). However, in triatomine cellular defense is performed by hemocytes, but these cells remains in the hemolymph and were never reported in the digestive tract lumen. Since, annexin are up-regulated in PM, one may expect that the microbiota is regulating the homeostasis of the antimicrobial function of NO in this digestive tract compartment. Interestingly, arginine is a substrate for NO production by NOS and cruzipain, a *T. cruzi* antigen, has been shown to induce arginase I expression in mouse ascites reticulum cell (Stempin et al., 2004). Moreover, *T. cruzi* is able to use this process to reduce NO production and to increase its population size in the TDT (Batista et al., 2020).

### Annexin Expression

*Serratia marcescens*, is a Gram-negative bacteria that compose *R. prolixus* microbiota (Azambuja et al., 2004; Vieira et al., 2016). Recently, it was identified that *S. marcescens* strains from *R. prolixus* gut presented many virulence factors (da Mota et al., 2019), as already described in other *S. marcescens* strains, including pore-forming toxins, such as hemolysins, which potentially cause damages to epithelial cells (Hertle, 2000, 2005; Ochieng et al., 2014; Lee et al., 2016). In *D. melanogaster*, it has been shown that *S. marcescens* infection induces gut damage and inflammatory reactions (Lee et al., 2016). In *R. prolixus*, *S. marcescens* population increase with blood ingestion and is more abundant in the seventh day after a blood meal in the anterior midgut, when compared to the posterior midgut (Vieira et al., 2016). It is possible that the microbiota proliferation could lead to inflammatory reactions in *R. prolixus* gut and could be related to the increase of annexins gene expression, observed in our analyses. Here we suggest a possible role of annexins in the maintenance of gut homeostasis, modulating anti-inflammatory responses, or acting as a signal to activate/deactivate other signaling cascades, such as the eicosanoid or JAK/STAT pathways. Although further experiments have to be done to assign annexin functions in triatomines correctly.

## Conclusion

The proteome analysis of the digestive tract from four triatomines species in the fifth nymphal stage revealed different proteins associated with those involved in fatty acid and carbohydrate metabolism indicating the priority of generating energy for triatomines at this stage. Other proteins, like cytochrome C oxidase, which participates in energy metabolism, also indicated a high degree of metabolic activity. The defense proteins identified, such as those associated with IMD, Toll, and JAK/STAT pathways and antimicrobial peptides, highlight the defensive function of the digestive tract of triatomines as an essential barrier for pathogen invaders. Furthermore, the presence of antioxidant proteins revealed that redox balance plays a significant role in this organ. Finally, annexin may play a role in the protection and homeostasis of intestinal epithelial cells against the inflammation induced by wounding compounds released during digestion, microbiota proliferation, and the release of effectors from the immune system. Since annexins are regulated by glucorticoids in vertebrates, it would be interesting to test how their level of expression may affect the microbiota and *T. cruzi* biology. Interestingly, annexins are up-regulated in PM compared to AM and their homeostasis in PM could be a contributing factor to the size of the *T. cruzi* population in the TDT. The results outlined here, along with previous data on genome, transcriptome and metabolome, allow a better understanding of the main factors and biological processes occurring in the triatomine digestive tract. This knowledge may help to uncover the interaction of *T. cruzi* with this system in an epidemiologic perspective.

## Data Availability Statement

We uploaded our proteome results on ProteomeXchange Consortium (http://proteomecentral.proteomexchange.org) under the accession numbers: PXD021625 for *D. maxima*, PXD021626 for *P. megistus*, PXD021627 *R. prolixus*, and PXD021628 for *T. infestans.*

## Author Contributions

MGu extracted the proteins from TDT, analyzed them by mass spectrometry (MS), and participated to the report writing. DM did the bioinformatics analyses. CV did the analyses of annexin expression and participated to the report writing. CSM prepared the primers for annexin analyses, sequenced annexin coding sequences, did the NJ tree, and participated to the report writing. CJM grew the different triatomine species. MSG participated to the report writing and reviewing. AT-F prepared the MS spectra. MW coordinated protein and MS analyses. PA participated in the result interpretation and report writing. MW, DM, and NC obtained grants to sustain the research. NC coordinated and participated to the bioinformatics analyses, he also coordinated the report writing. All authors contributed to the article and approved the submitted version.

## Conflict of Interest

The authors declare that the research was conducted in the absence of any commercial or financial relationships that could be construed as a potential conflict of interest.

## Footnotes

1 https://www.uniprot.org/proteomes/query=hemiptera

2 www.massive.org.au

3 http://proteomecentral.proteomexchange.org

4 www.vectorbase.org

5 https://www.genome.jp/kegg/pathway.html

6 https://www.ncbi.nlm.nih.gov/protein/

7 https://www.uniprot.org/

8 http://ensemblgenomes.org/

9 http://www.cobea.org.br/

## Supplementary Material

Supplementary File 1 Cumulated list of non-redundant protein acessions from this work, Ribeiro et al. (2014), Vieira et al. (2015), and Ouali et al. (2020). SF1a – Complete UniprotKB list. SF1b – List from 1a reduced to the accessions associated to EC numbers.

Supplementary File 2 Ontologies of *Biological Processes* analyzed with GlueGO using data from Supplementary File SF1a. SF2a – Simplified topological network generated from a dataset of 22,291 edges based on 2,569 vertices. SF2b – Frequency histogram of biological processes in the TDT proteome. SF2c – Pie statistical display of SF2b. SF2d – Numerical data supporting SF2a–c. The color code associated to vertices of SF2a is the same as for SF2b and SF2c.

Supplementary File 3 Ontologies of *Cellular Components* analyzed with GlueGO using data from Supplementary File SF1a. SF3a – Simplified topological network generated from a dataset of 6,405 edges based on 1,306 vertices. SF3b – Frequency histogram of cellular components in the TDT. SF3c – Pie statistical display of SF3b. SF3d – Numerical data supporting SF3a–c. The color code associated to vertices of SF3a is the same as for SF3b and SF3c.

Supplementary File 4 Ontologies of *Molecular Functions* analyzed with GlueGO using data from Supplementary File SF1a. SF4a – Simplified topological network generated from a dataset of 2,087 edges based on 795 vertices. SF4b – Frequency histogram of molecular functions in the TDT. SF4c – Pie statistical display of SF4b. SF4d – Numerical data supporting SF4a-c. The color code associated to vertices of SF4a is the same as for SF4b and SF4c.

Supplementary File 5 Ontologies of *Biological Processes* analyzed with GlueGO using data from Supplementary File SF1b. SF5a – Simplified topological network generated from a dataset of 8,756 edges based on 703 vertices. SF5b – Frequency histogram of biological processes in the TDT proteome. SF5c – Pie statistical display of SF5b. SF5d – Numerical data supporting SF5a-c. The color code associated to vertices of SF5a is the same as for SF5b and SF5c.

Supplementary File 6 Ontologies of *Cellular Components* analyzed with GlueGO using data from Supplementary File SF1b. SF6a – Simplified topological network generated from a dataset of 798 edges based on 175 vertices. SF6b – Frequency histogram of cellular components in the TDT. SF6c – Pie statistical display of SF6b. SF6d – Numerical data supporting SF6a–c. The color code associated to vertices of SF6a is the same as for SF6b and SF6c.

Supplementary File 7 Ontologies of *Molecular Functions* analyzed with GlueGO using data from Supplementary File SF1b. SF7a – Simplified topological network generated from a dataset of 3,044 edges based on 383 vertices. SF7b – Frequency histogram of molecular functions in the TDT. SF7c – Pie statistical display of SF7b. SF7d – Numerical data supporting SF7a–c. The color code associated to vertices of SF7a is the same as for SF7b and SF7c.

Supplementary File 8 Spreadsheet of similarity matches from triatomines’ enzymes. The sequences of our samples matching UniprotKB and the sequences from the datasets of *R. prolixus* from Ribeiro et al. (2014), Vieira et al. (2015) and Ouali et al. (2020) retrieved from VectorBase and ProteomeXchange were annotated for their enzymatic function (EC number) by reference to KEGG using BLASTp.

Supplementary File 9 KEGG map of Citrate Cycle pathway after excluding alternative routes. Adapted from KEGG (https://www.genome.jp/kegg/). The sequences from our samples matching UniprotKB (yellow) and the sequences from Ribeiro et al. (2014), Vieira et al. (2015), or Ouali et al. (2020) (blue) retrieved from VectorBase and ProteomeXchange were annotated for their enzymatic function (EC number) by reference to KEGG using BLASTp. ECs on blue background were absent from our samples data. ECs on red background were not present in all four sources.

Supplementary File 10 KEGG map of Fatty Acid Elongation pathway after excluding alternative routes. Adapted from KEGG (https://www.genome.jp/kegg/). The sequences from our samples matching UniprotKB (yellow) and the sequences from Ribeiro et al. (2014) or Ouali et al. (2020) (blue) retrieved from VectorBase and ProteomeXchange were annotated for their enzymatic function (EC number) by reference to KEGG using BLASTp. ECs on blue background were absent from our samples data.

Supplementary File 11 KEGG map of Fatty Acid Degradation pathway after excluding alternative routes. Adapted from KEGG (https://www.genome.jp/kegg/). The sequences from our samples matching UniprotKB (yellow) and the sequences from Ribeiro et al. (2014) or Ouali et al. (2020) (blue) retrieved from VectorBase and ProteomeXchange were annotated for their enzymatic function (EC number) by reference to KEGG using BLASTp. ECs on blue background were absent from our samples data.

Supplementary File 12 KEGG map of Glycolysis/Gluconeogenesis pathway after excluding alternative routes. Adapted from KEGG (https://www.genome.jp/kegg/). The sequences from our samples matching UniprotKB (yellow) and the sequences from Ribeiro et al. (2014), Vieira et al. (2015), or Ouali et al. (2020) (blue) retrieved from VectorBase and ProteomeXchange were annotated for their enzymatic function (EC number) by reference to KEGG using BLASTp. ECs on blue background were absent from our samples data. ECs on red background were not present in all four sources.

Supplementary File 13 KEGG map of Oxidative Phosphorylation pathway after excluding alternative routes. Adapted from KEGG (https://www.genome.jp/kegg/). The sequences from our samples matching UniprotKB (yellow) and the sequences from Ribeiro et al. (2014), Vieira et al. (2015), or Ouali et al. (2020) (blue) retrieved from VectorBase and ProteomeXchange were annotated for their enzymatic function (EC number) by reference to KEGG using BLASTp. ECs on blue background were absent from our samples data. ECs on red background were not present in all four sources.

Supplementary File 14 Spreadsheet of similarity matches from triatomines’ innate immune system. The protein sequences of our samples and those from the datasets of *R. prolixus* from Ribeiro et al. (2014), Vieira et al. (2015) and Ouali et al. (2020) retrieved from VectorBase and ProteomeXchange were annotated by reference to IIID, the immune pathways from KEGG (code: 04624 and 04013), insects’ PGRP proteins, RPRC003519, RPRC011897 and RPRC013832 sequences from NCBI, using BLASTp.
