# Supplementary figures and images for: Proteome of the Triatomine Digestive Tract: From Catalytic to Immune Pathways; Focusing on Annexin Expression
Source: Front Mol Biosci. 2020 Dec 9;7:589435. doi: 10.3389/fmolb.2020.589435 (PMC7755933; doi:10.3389/fmolb.2020.589435)

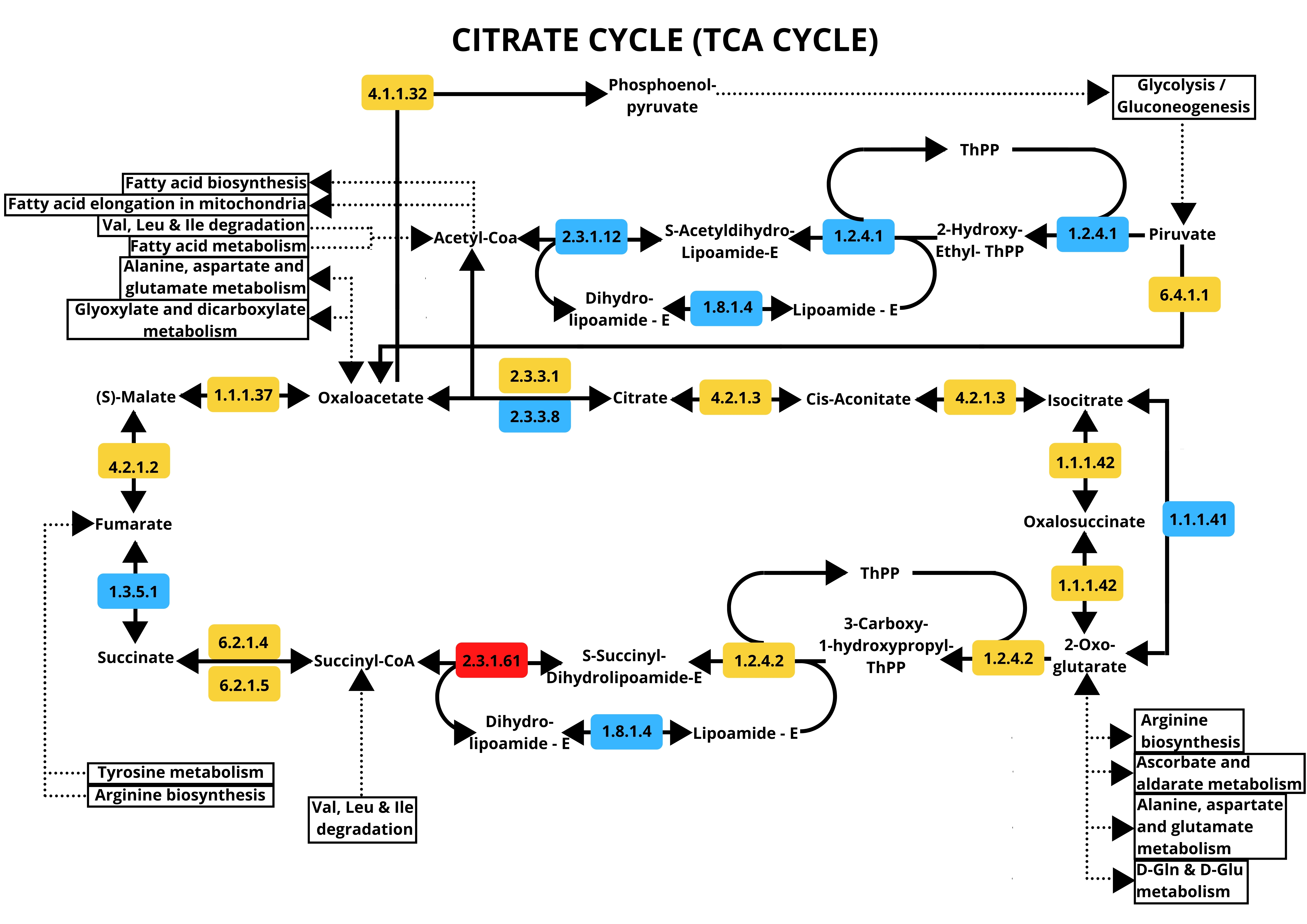

Supplement: Supplementary File 9 — KEGG map of Citrate Cycle pathway after excluding alternative routes. Adapted from KEGG (https://www.genome.jp/kegg/). The sequences from our samples matching UniprotKB (yellow) and the sequences from Ribeiro et al. (2014), Vieira et al. (2015), or Ouali et al. (2020) (blue) retrieved from VectorBase and ProteomeXchange were annotated for their enzymatic function (EC number) by reference to KEGG using BLASTp. ECs on blue background were absent from our samples data. ECs on red background were not present in all four sources. [file Image_1.JPEG]

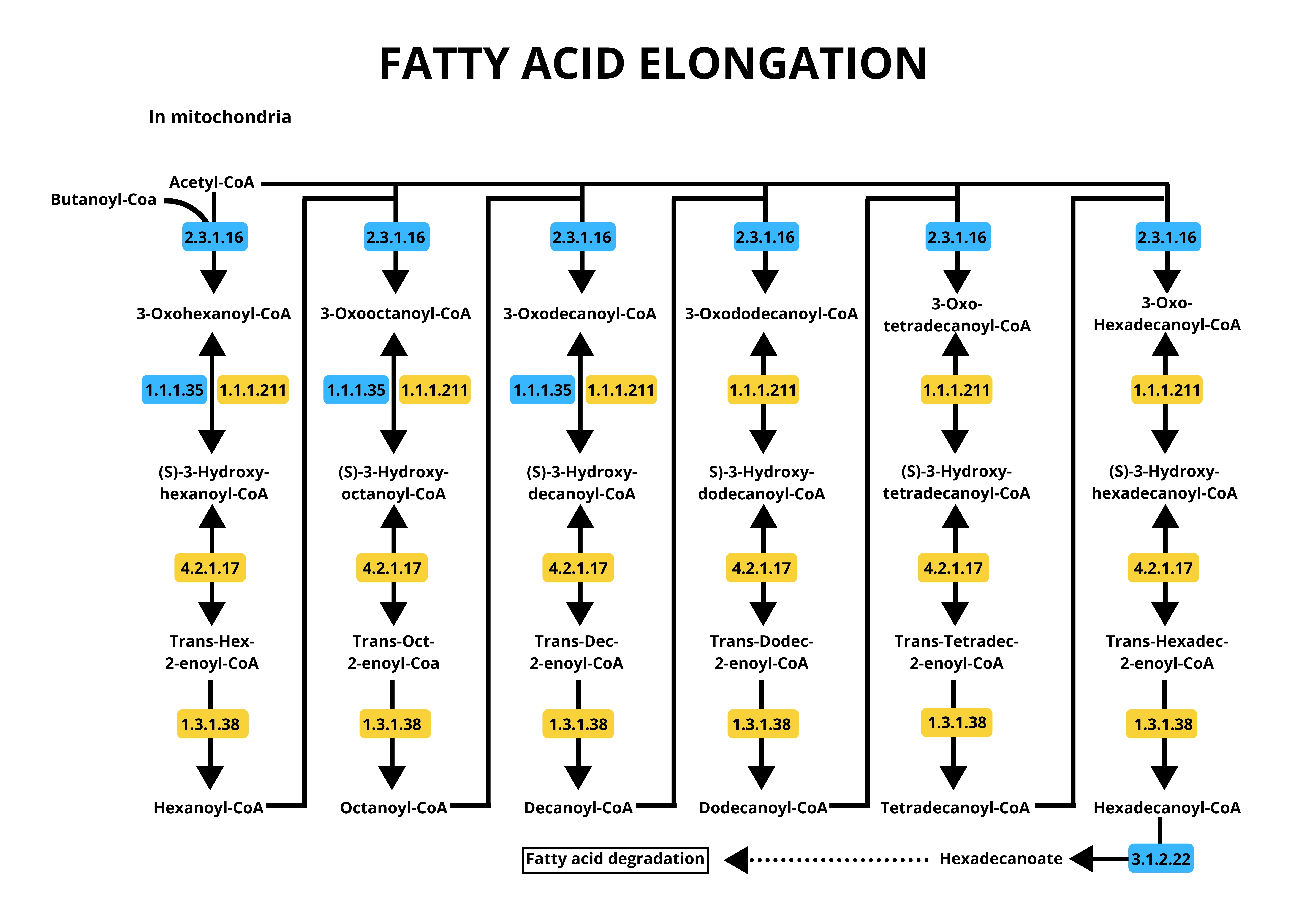

Supplement: Supplementary File 10 — KEGG map of Fatty Acid Elongation pathway after excluding alternative routes. Adapted from KEGG (https://www.genome.jp/kegg/). The sequences from our samples matching UniprotKB (yellow) and the sequences from Ribeiro et al. (2014) or Ouali et al. (2020) (blue) retrieved from VectorBase and ProteomeXchange were annotated for their enzymatic function (EC number) by reference to KEGG using BLASTp. ECs on blue background were absent from our samples data. [file Image_2.JPEG]

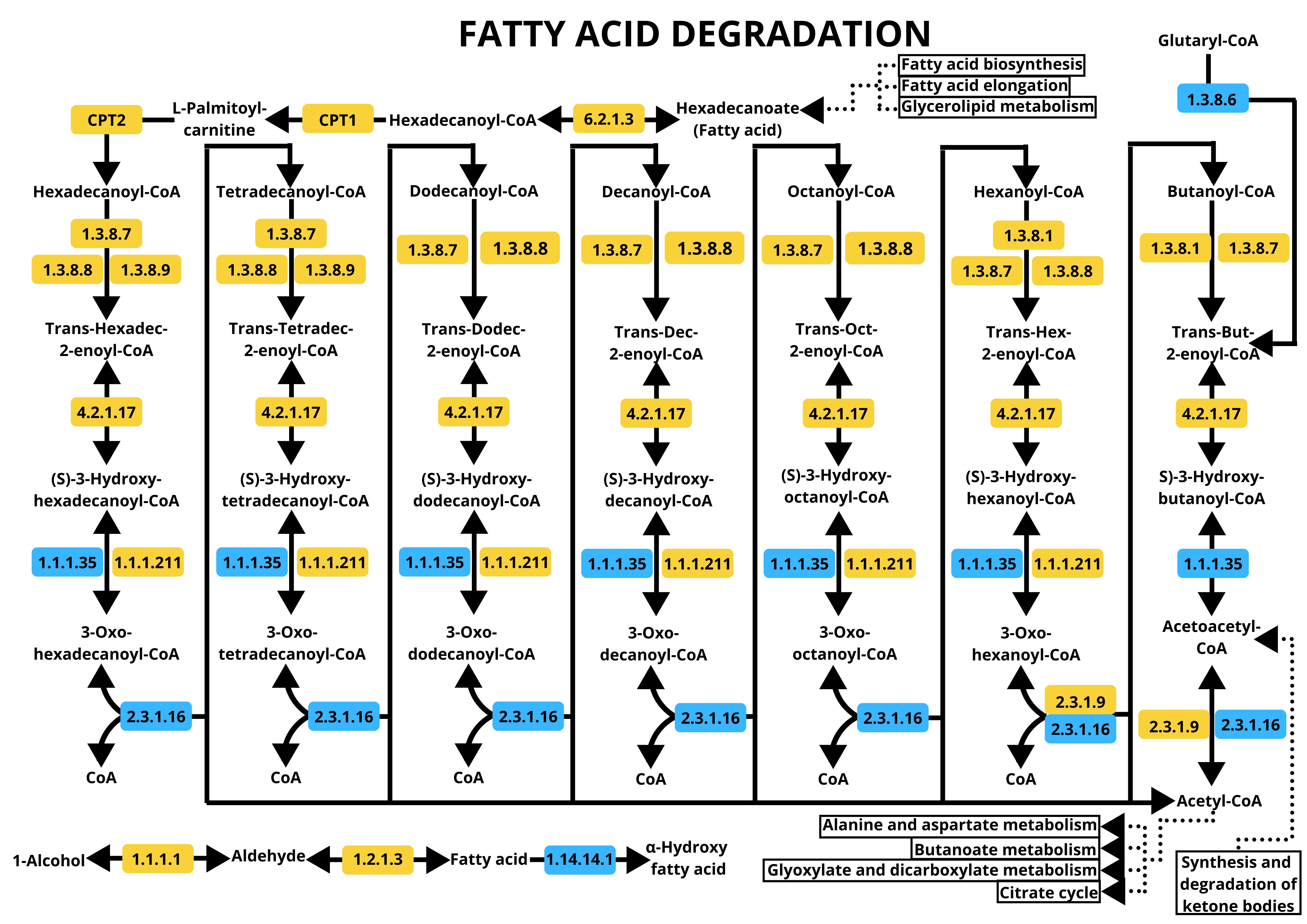

Supplement: Supplementary File 11 — KEGG map of Fatty Acid Degradation pathway after excluding alternative routes. Adapted from KEGG (https://www.genome.jp/kegg/). The sequences from our samples matching UniprotKB (yellow) and the sequences from Ribeiro et al. (2014) or Ouali et al. (2020) (blue) retrieved from VectorBase and ProteomeXchange were annotated for their enzymatic function (EC number) by reference to KEGG using BLASTp. ECs on blue background were absent from our samples data. [file Image_3.JPEG]

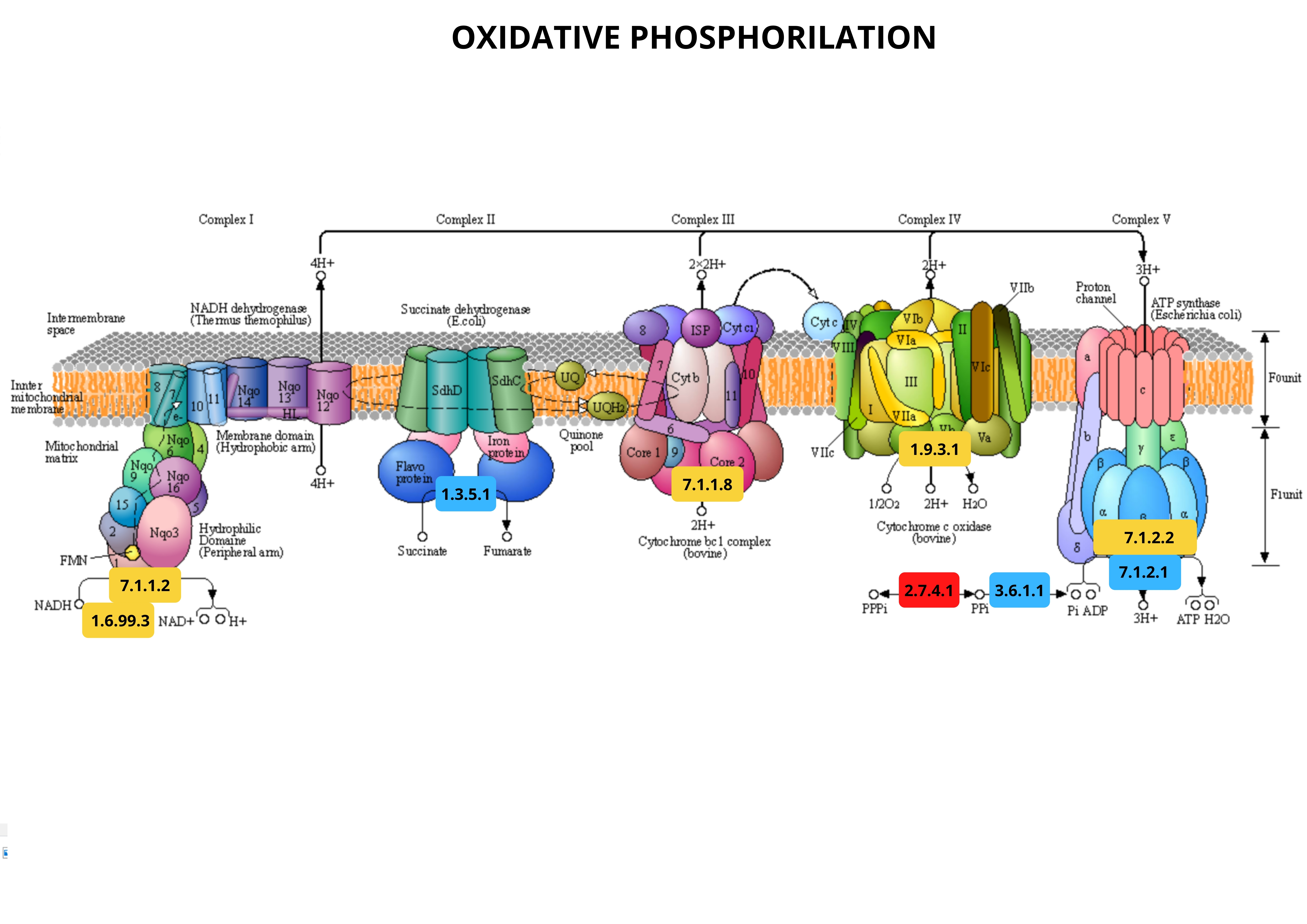

Supplement: Supplementary File 13 — KEGG map of Oxidative Phosphorylation pathway after excluding alternative routes. Adapted from KEGG (https://www.genome.jp/kegg/). The sequences from our samples matching UniprotKB (yellow) and the sequences from Ribeiro et al. (2014), Vieira et al. (2015), or Ouali et al. (2020) (blue) retrieved from VectorBase and ProteomeXchange were annotated for their enzymatic function (EC number) by reference to KEGG using BLASTp. ECs on blue background were absent from our samples data. ECs on red background were not present in all four sources. [file Image_5.JPEG]
